# Design Principles for Deployable Fibers Inspired by Hagfish Defense

**DOI:** 10.1002/advs.202512414

**Published:** 2025-10-07

**Authors:** Mohammad Tanver Hossain, Wonsik Eom, Pallab Layak, Jeongmin Kim, Carolyn Darling, Andrew Lowe, Douglas S. Fudge, Sameh H. Tawfick, Randy H. Ewoldt

**Affiliations:** ^1^ Department of Mechanical Science and Engineering Grainger College of Engineering University of Illinois at Urbana‐Champaign Urbana IL 61801 USA; ^2^ Beckman Institute for Advanced Science and Technology University of Illinois at Urbana‐Champaign Urbana IL 61801 USA; ^3^ Department of Fiber Convergence Material Engineering Dankook University Yongin‐si Republic of Korea; ^4^ Schmid College of Science and Technology Chapman University Orange 92866 USA

**Keywords:** applied mechanics, bioinspired design, defense mechanism, engineering design, hagfish slime, soft matter

## Abstract

Hagfish produce extraordinary slime as a defense mechanism, releasing exudate from glands that rapidly form a fibrous, soft, ultra‐dilute, water‐capturing network upon contact with seawater (up to 10 000 times its original volume). The gland thread cell (GTC) produces high‐strength protein threads (filament diameter *d*_*f*_ = 1–3 µm) meticulously coiled into skeins (coil diameter *D*_*o*_ ∼ 150 µm) that rapidly unravel upon deployment to reveal their hidden length (*L*_*f*_ = 15 cm), forming a cohesive fibrous slime network through interaction with mucin vesicles and seawater. To date, no engineered material is able to replicate the fiber uncoiling mechanics observed in slime, which are responsible for the unique set of mechanical properties that slime exhibits. Focusing on fundamental physical mechanisms rather than specific biochemistry or biomaterials, it is demonstrated that how existing materials and manufacturing processes can be used to achieve comparable functional performance. To engineer rapidly deployable soft materials inspired by hagfish slime, this work establishes design principles for synthetic skeins used to create the first‐ever deployable synthetic skeins. Four design principles are revealed for engineering synthetic skeins: (1) the mechanics of high‐strain fiber coiling and uncoiling, (2) adhesives to maintain elastic energy in non‐equilibrium deformed states, (3) fluid‐mediated deployment of coiled fibers, and (4) the individual fiber stiffness and size needed to result in a soft, deformable fibrous network. As proof of concept, the first successful fabrication of synthetic skeins with tightly coiled threads arranged in controlled packing geometries is demonstrated. These synthetic structures undergo fluid‐mediated unraveling under flow, replicating the deployment behavior of their biological counterparts and demonstrating the feasibility of engineered, deployable fibrous networks.

## Graphical abstract

Hagfish slime deploys fibers from coiled skeins to form an ultra‐dilute, water‐trapping network. Inspired by this mechanism, design principles are established for synthetic skeins, fabricated with tightly coiled fibers that unravel under flow. These synthetic skeins demonstrate deployable fibrous networks, highlighting generalizable strategies for rapid, compact‐to‐extended transitions in soft, functional materials.

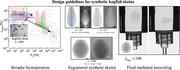

## 1 Introduction

**Figure 1:**
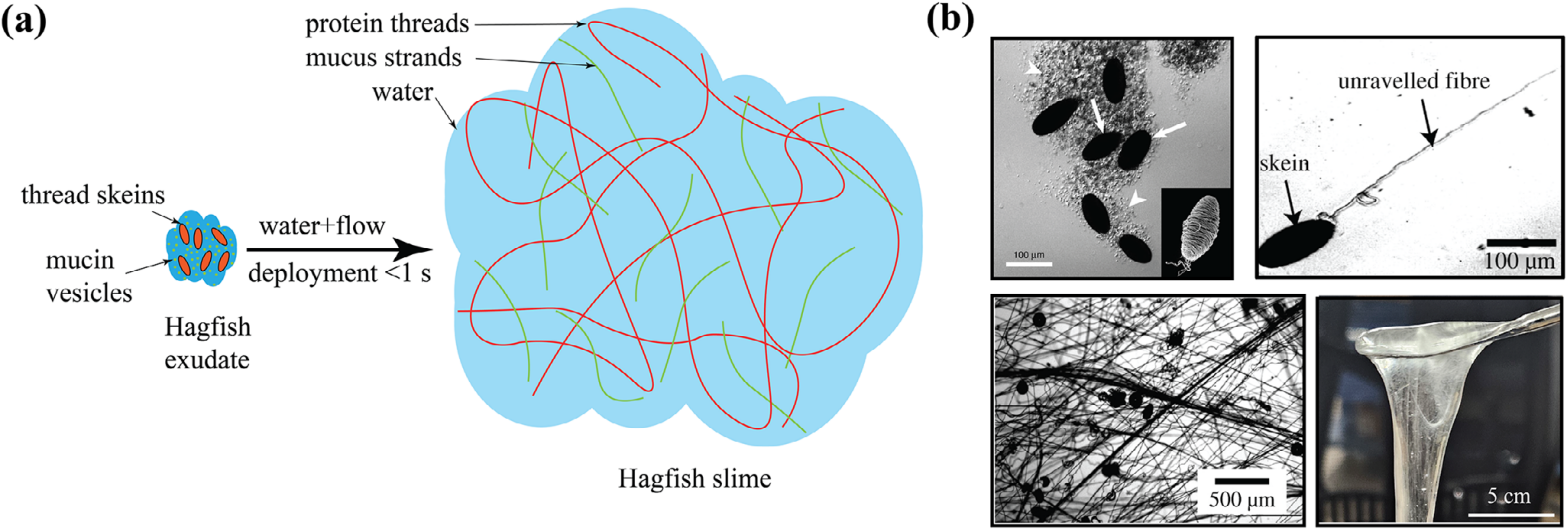
a) Hagfish slime gland exudate contains two main solid components ‐ thread skeins that consist of a single coiled intermediate filament bundle, and mucin vesicles. A skein unravels rapidly under the hydrodynamic forces from the surrounding flow field. The unraveled threads and mucus entrain a large volume of water to form a cohesive network (adapted with permission from Hossain et al. (2025)^[^ ^15^ ^]^). b) From upper left going clockwise: Optical image of hagfish exudate showing thread skeins (arrows), mucin vesicles (arrowheads), and inset of thread in its initial conical loop arrangement (height of the skein is 120 µm) (reused with permission from Herr et al. (2010)^[^ ^7^ ^]^ and Knight (2019)^[^ ^19^ ^]^). A single skein unraveled to expose the hidden length (reused with permission from Chaudhary et al. (2019)^[^ ^10^ ^]^). Image of hagfish slime produced by mixing hagfish exudate with seawater (reused with permission from Hossain et al. (2025)^[^ ^15^ ^]^). Microscope image of the slime showing the unraveled fibers (reused with permission from Chaudhary et al. (2019)^[^ ^10^ ^]^).

Nature often unveils solutions to engineering challenges in the most unexpected ways, and the hagfish—an ancient, eel‐like marine creature—is a prime example. The hagfish is renowned for its ability to secrete a unique slime that acts as an effective defense mechanism by clogging the gills of predators.^[^ ^1^, ^2^, ^3^, ^4^, ^5^, ^6^ ^]^ Hagfish slime is an extraordinary material formed from glandular exudates that rapidly expand into a cohesive and elastic hydrogel upon contact with seawater. This defense mechanism involves ejecting a small amount of biomaterial (exudate) into the water, which expands up to 10 000 times its original volume to form a cohesive, mucus‐like mass, capable of potentially choking predators^[^ ^3^ ^]^ (**Figure** **1**a). This unique property makes hagfish slime the most dilute hydrogel known and an unparalleled example of fluid‐structure interaction. The slime consists of two primary components: mucus produced by gland mucous cells and fibrous threads produced by gland thread cells (GTC) (Figure 1b).

The GTC is a marvel of biology and a key to the hagfish's survival. It produces protein threads (diameter *d*_*f*_ = 1 − 3 µm) of exceptional strength (∼180 MPa), stiffness (*E*_*f*_ ∼ 6.4 MPa), and length (*L*_*f*_ ∼ 15 cm), which are meticulously coiled into conical loop arrangements (skeins) within the cytoplasm^[^ ^2^, ^7^, ^8^, ^9^, ^10^, ^11^, ^12^, ^13^, ^14^, ^15^, ^16^, ^17^ ^]^ (Figure 1b). Upon deployment, these threads unravel, interacting with mucin vesicles and seawater to create the slime's cohesive network^[^ ^2^, ^10^, ^18^ ^]^ (Figure 1b). This unraveling process occurs rapidly, aligning with the timescale of a predator attack, taking place within 0.4 seconds.^[^ ^10^ ^]^ The resulting fibrous network forms a soft solid material with a linear elastic shear modulus of *G*′ ∼ 0.02 Pa for timescales of 0.1 s ⩽*t* ⩽ 10 s, making it one of the softest biomaterials known.^[^ ^5^ ^]^ Under extensional flow, hagfish slime stiffens into a gill‐blocking mesh, while under shear flow, it rapidly collapses to prevent self‐entanglement.^[^ ^14^ ^]^

Currently, no synthetic material possesses the unique physical properties of hagfish slime, particularly the mechanism of unraveling coiled fibers to form a fibrous water‐capturing network. In nature, thread skeins exhibit an extraordinary hidden length ratio of approximately 1:1000, uncoiling from an initial size of about ∼150 µm to a length of ∼15 cm.^[^ ^2^ ^]^ Current state‐of‐the‐art superabsorbent materials (SAMs) exhibit a swelling capacity of up to 3000 times their mass in seawater, with more typical SAMs achieving swelling factors of around 300.^[^ ^20^ ^]^ In stark contrast, a small quantity of material (∼40 mg dry weight) ejected from hagfish slime glands into seawater can transiently generate approximately 1 kg of slime, representing a remarkable 25 000‐fold mass increase.^[^ ^2^, ^15^ ^]^ This is particularly impressive given that the swelling capacity of SAMs in saline solutions is typically reduced by one to two orders of magnitude.^[^ ^21^ ^]^

It remains an open question whether precisely mimicking the exact properties of hagfish thread skeins is necessary for fabricating deployed synthetic fiber‐based water‐capturing materials. Mimicking specific elements of hagfish slime, especially the unique hagfish threads, presents substantial technical challenges due to their inherent complexity. Is it necessary to precisely replicate the dimensions and mechanical characteristics of natural skeins, e.g. could larger or stiffer threads achieve the same functional behavior? Here, we propose a bioinspired design perspective that seeks to replicate the functional deployment behavior of hagfish slime threads without requiring an exact match to the natural thread dimensions or mechanics.^[^ ^23^ ^]^

**Figure 2:**
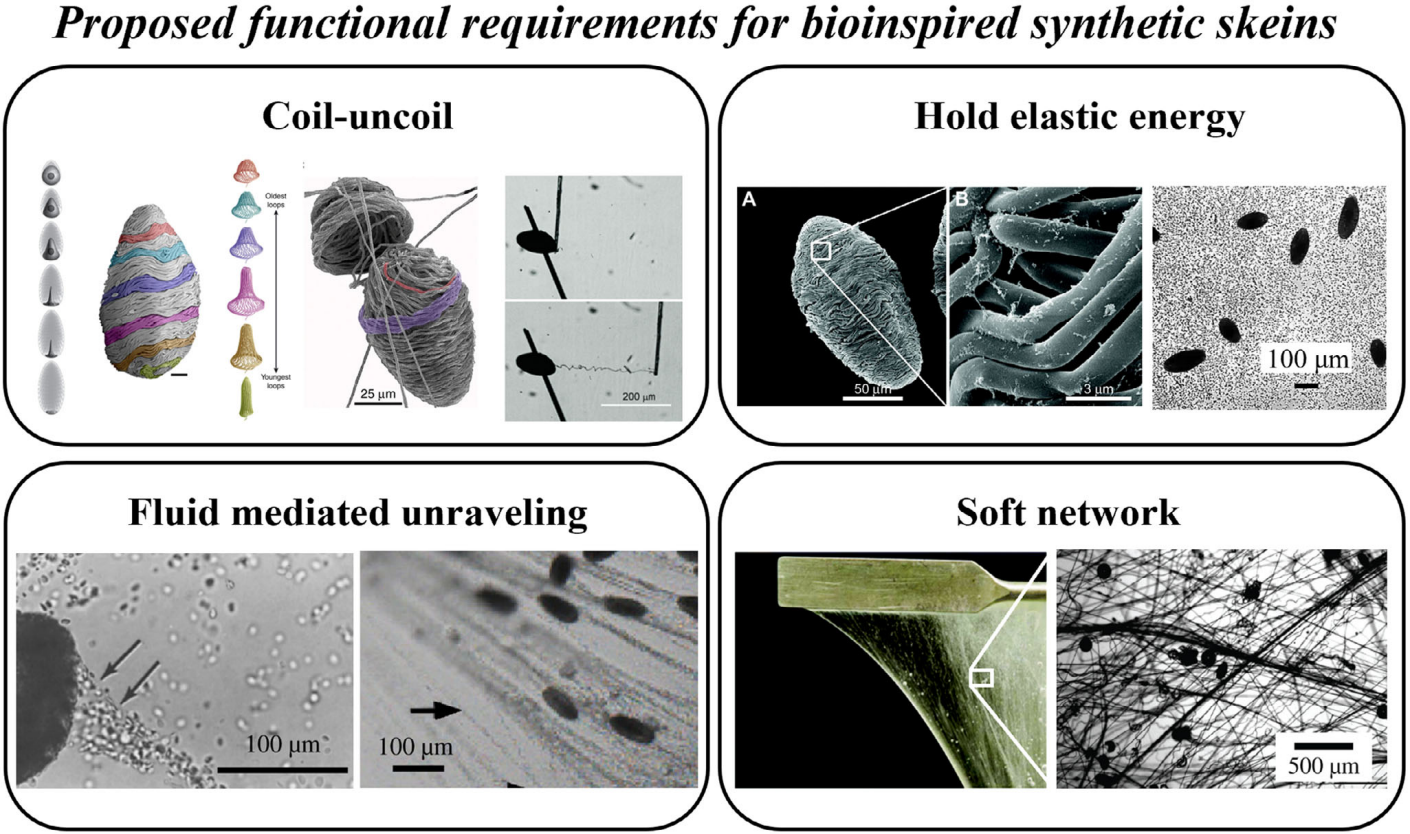
Proposed four functional requirements for synthetic skeins to replicate hagfish slime behavior. From upper left going clockwise: Coil‐uncoil transition to reveal a significant hidden length (adapted from Winegard et al. (2014)^[^ ^12^ ^]^ and Hossain et al. (2025)^[^ ^15^ ^]^), hold elastic energy in the non‐equilibrium state before unraveling (reused with permission from Bernards et al. (2014)^[^ ^6^ ^]^), form a soft fibrous network after deployment (adapted from Rementzi et al. (2019)^[^ ^22^ ^]^ and Chaudhary et al. (2019)^[^ ^10^ ^]^), and deployment in flow (reused with permission from Chaudhary et al. (2019) ^[^ ^10^ ^]^).

Observations of natural skeins and the slime formation process (Figure 1) lead us to identify four functional requirements associated with the hagfish slime formation (**Figure** **2**). First, the skeins undergo a coil‐uncoil transition to reveal a hidden length far greater than their coiled size. Second, elastic energy may be retained in a non‐equilibrium state, either before unraveling if the fiber's equilibrium is uncoiled or after unraveling if the equilibrium is coiled. Third, the transition from coil to uncoil occurs rapidly under fluid flow, facilitating swift network formation. Fourth, a collection of uncoiled fibers forms a soft, fibrous network after deployment, capable of undergoing large deformations without failing. Multiple engineering strategies could address these requirements, including controlling the equilibrium state of the skeins (coiled or uncoiled) and leveraging manufacturing methods such as embedded 3D printing ^[^ ^24^, ^25^, ^26^, ^27^, ^28^, ^29^ ^]^ in yield‐stress fluids.

Here, we study how to achieve these functional requirements from an applied mechanics perspective, developing mathematical scaling relations, approximate analytical models, and resulting design principles, and ultimately the first‐ever creation of synthetic deployable thread skeins. Hagfish skeins are hierarchically assembled from proteins to intermediate filaments (∼10 nm) to skein threads (1–3 µm). Our focus is on thread‐level properties, independent of or agnostic to the material chemistry of the thread composition. We are not aiming to imitate synthetic proteins; instead, we aim to replicate larger‐scale fibers that can achieve the functional properties of deployability and network formation. Our bioinspired engineering approach integrates principles from solid mechanics, fluid mechanics, rheology, and soft matter physics. Although no synthetic material currently replicates the extraordinary properties of hagfish slime, by focusing on fundamental physical mechanisms rather than specific biochemistry or biomaterials, we demonstrate how existing materials and manufacturing processes can be used to achieve comparable functional performance. Using the design framework, we fabricate synthetic skeins using the recently developed method of 3D printing by solvent exchange (3DPX);^[^ ^27^ ^]^ we demonstrate complex coil topology with significant hidden length that unravels in flow without tangling or breaking, replicating functional requirements of the natural system.

## 2 Principles of Tight Elastic Packing and Uncoiling

Hagfish slime contains thousands of intermediate filament threads (*d*_*f*_ ≈ 1–3 µm) packed into ∼150 µm skeins that unravel to ∼15 cm lengths.^[^ ^12^ ^]^ Engineering an analog requires coiling threads into skeins and uncoiling them during deployment to achieve the target hidden length.

One challenge lies in avoiding material failure while undergoing significant strain during the coil‐uncoil transition. This requires addressing constraints related to packing density, material strain‐to‐break limit (ϵ_break_), fiber diameter (*d*_*f*_), and overall packing diameter (*D*_*o*_). Successfully engineering such a system demands a balance between geometric and material properties.

A key question is whether replicating the exact properties of hagfish threads is necessary or if a broader design framework can achieve similar functionality. By densely packing thin, deformable fibers, we expect to replicate the performance of hagfish threads; however, thicker soft fibers or thinner, less deformable fibers may also suffice, and we analyze the design trade‐offs between fiber size, packing density, and strain limits.

Here, we quantify the strain (ϵ) associated with the coil‐uncoil transition required to achieve a hidden length ratio (λ) for a nested cylindrical coil (Section 2.1) and complex topologies (section 2.2) as a function of relevant parameters such as fiber diameter (*d*_*f*_), packing diameter (*D*_*o*_), and infill void ratio (2ℜ_min _/*D*_*o*_), which determines how compactly the thread must be packed to achieve the desired hidden length. The resulting framework is versatile and can be extended to other topologies, providing a foundation for a wide range of coil‐uncoil transition systems beyond the biomimetic conditions.

### 2.1 Results: Cylindrical Coil Topology

**Figure 3:**
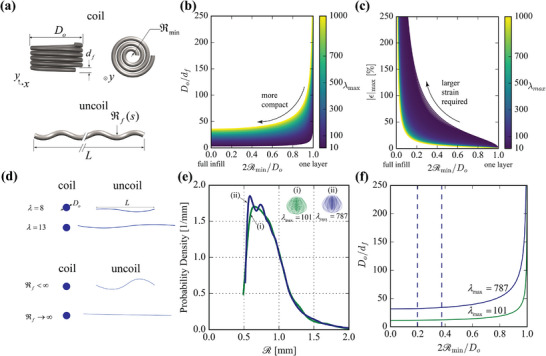
Principles of tight elastic packing and uncoiling. a) Schematic of a nested cylindrical coil topology with outer diameter *D*_*o*_, thread diameter *d*_*f*_, and minimum radius of curvature ℜ_min _. After uncoiling, it reveals a total end‐to‐end length *L* with a final radius of curvature distribution ℜ_*f*_(*s*). b) Feature size ratio *D*_*o*_/*d*_*f*_ as a function of infill void ratio 2ℜ_min _/*D*_*o*_ (coil tightness) and hidden length ratio λ_max _ for cylindrical coils. More infill (lower 2ℜ_min _/*D*_*o*_) enables more compact *D*_*o*_/*d*_*f*_ for a given hidden length ratio λ_max _. c) Maximum strain required for full coil‐uncoil. Tighter coiling necessitates larger strains. d) Schematic showing concepts of different λ_max _ and ϵ_max _. e) Curvature distribution for two complex topologies. Insets illustrate the skein configurations with distinct hidden length ratios. f) Feature size ratio *D*_*o*_/*d*_*f*_ for the two complex topologies as a function of infill void ratio 2ℜ_min _/*D*_*o*_, with vertical lines representing the range of void ratios corresponding to the most frequently observed curvature values in panel (e).

Consider a simple case where the thread is arranged in a nested cylindrical coil topology, characterized by its largest feature size, *D*_*o*_ (e.g., the coil's outer diameter), and a total fiber length, *L*_*f*_, which, when fully uncoiled, spans an approximate end‐to‐end distance *L* (**Figure** **3**a). Fully uncoiled means that the fiber is geometrically straightened relative to its initial coiled configuration, independent of whether elastic energy or stresses remain. *D*_*o*_ is directly related to the fabrication process, representing the maximum allowable diameter constrained by the available space or volume within the fabrication method. This constraint influences how fibers can be packed within the skein‐like structure and determines the achievable hidden length upon uncoiling. This simplified model helps identify the critical parameters that must be addressed, even for more complex geometries.

The key parameter associated with the coil–uncoil functional requirements is the hidden length ratio, λ, defined as the ratio of uncoiled to coiled size: $\lambda =\frac{L}{D_{o}}$. The maximum hidden length ratio is $\lambda_{\max}=\frac{L_{f}}{D_{o}}$, which corresponds to the case where the fiber is completely straightened. Another important parameter is the feature size ratio, *D*_*o*_/*d*_*f*_, which compares the coil diameter *D*_*o*_ to the fiber diameter *d*_*f*_. The feature size ratio is related to the fabrication process, as fabrication is likely more difficult at higher values of *D*_*o*_/*d*_*f*_, i.e. difficulty increases to fabricate a smaller fiber diameter *d*_*f*_ for the same overall build size *D*_*o*_.

Consider a single fiber arranged on nested cylindrical paths where the outer diameter of the cylinder is *D*_*o*_ and the height of the coiled cylinder is *H* = *D*_*o*_ (the aspect ratio of one, a nominally compact topology). For a given outer diameter *D*_*o*_, more fiber length can be included if the inner void is filled, requiring a coil tightness with local radius of curvature ℜ < *D*_*o*_/2. With this nested cylinder topology (Figure 3a), the fiber fills the inner void to an inner diameter *D*_*i*_, which is related to the minimum radius of curvature of the fiber, ℜ_min _ = *D*_*i*_/2. The total fiber length *L*_*f*_ relates to the number of layers *n* along the height and the number of nested loops *m* per layer, which gives *L*_*f*_ = *mn*π(*D*_*o*_ − *md*_*f*_) in the limit of *d*_*f*_ ≪ *D*_*o*_. Relating *n* and *m* to geometric parameters, where *n* = *H*/*d*_*f*_ = *D*_*o*_/*d*_*f*_ and *m* = (*D*_*o*_/2 − ℜ_min _)/*d*_*f*_, substituting into the hidden length ratio as λ_max _ = *L*_*f*_/*D*_*o*_, and grouping into dimensionless terms gives

(1) $$\lambda_{\max}=\frac{\pi }{4}{\left(\frac{D_{o}}{d_{f}}\right)}^{2}\left\{1-{\left(\frac{{ℜ}_{\min}}{D_{o}/2}\right)}^{2}\right\}.$$

Key dimensionless parameters are revealed by Equation 1, including *D*_*o*_/*d*_*f*_ and the ratio of the coil tightness to the outer diameter, 2ℜ_min _/*D*_*o*_, which can be interpreted as the infill void fraction, since the amount of void goes to zero (fully‐filled) as ℜ_min _ → 0. Figure 3b represents the tradeoffs between *D*_*o*_/*d*_*f*_ and 2ℜ_min _/*D*_*o*_. Decreasing the void amount enables more compact *D*_*o*_/*d*_*f*_ for a given λ. However, although ℜ_min _ → 0 maximizes λ_max _ to $\lambda_{\max}^{0}=\left(\pi /4\right){\left(D_{o}/d_{f}\right)}^{2}$, it may be physically unachievable since it would require infinite strain to uncoil and straighten a fiber with zero radius of curvature. Synthetic skein architecture generated from nested cylindrical coils (λ_max _ = 113, *H* = *D*_*o*_ = 6 mm) is shown in Figure S1a (Supporting Information).

During the coil–uncoil transition, the maximum strain ϵ_max _ must remain below the material strain‐to‐break limit ϵ_break_ to allow full uncoiling without fracture. Using classical beam bending kinematics, the maximum tensile or compression strain generated when a fiber transitions from an initial radius of curvature ℜ_min _ to a new curvature ℜ_*f*_ is given by

(2) $$\left|\varepsilon \right|=\frac{d_{f}}{2}\left(\frac{1}{{ℜ}_{\min}}-\frac{1}{{ℜ}_{f}}\right).$$

This relation describes the value of the strain at the outermost radius by assuming small‐strain, linear‐elastic deformation and applies to fibers with circular cross‐sections under the assumption that ℜ_min _ ≫ *d*_*f*_. To fully straighten, ℜ_*f*_ → ∞, the maximum strain becomes

(3) $${\left|\varepsilon \right|}_{\max}=\frac{d_{f}/2}{{ℜ}_{\min}}.$$

Figure 3c illustrates the relationship between |ϵ|_max _ and the infill void ratio, showing that strain increases and approaches infinity as the void ratio approaches zero (fully‐filled configuration). While |ϵ|_max _ appears inversely related to λ, this is because for a given curvature, higher hidden length requires thinner fibers, which experience less strain for the same change in curvature. This formulation assumes that the fiber behaves as a slender elastic beam, where bending dominates over stretching, and is valid under the condition ℜ ≫ *d*_*f*_. While the assumption breaks down in the extreme case of ℜ_min _ → 0, however for *d*_*f*_ ≪ *D*_*o*_, the fraction of fiber length experiencing such tight curvatures is small relative to the total fiber length. Thus, despite its limitations, this beam‐based strain model provides a physically reasonable and conservative estimate of the maximum strain during the coil–uncoil transition for the majority of the fiber.

Figure 3d shows a schematic with the same outer coil diameter but different hidden length ratios and varying strain during uncoiling. By specifying a target λ and *D*_*o*_/*d*_*f*_ from a manufacturing standpoint, the required 2ℜ_min _/*D*_0_ (coil tightness) can be approximated using Equation 1 and the resulting |ϵ|_max _ in the thread using Equation 3. Conversely, given a material with known strain‐to‐break ϵ_break_, targeted λ, and *D*_*o*_, the required *d*_*f*_ and ℜ_*min*_ can be obtained. These parameters form the basis for establishing design guidelines, providing insight into how to balance material properties and geometric constraints to achieve the desired performance. Moreover, the principles derived from this analysis are generalizable as we will see in the next section, enabling their application to more complex topologies and informing the engineering of synthetic skeins across diverse configurations.

### 2.2 Results: Complex Topology

We apply the framework established in the previous section to a complex topology as shown in the inset of Figure 3e and in Figure S1b,c (Supporting Information). Inspired by natural skeins,^[^ ^12^ ^]^ the topology is generated using a combination of parametric equations and numerical interpolation to define helical structures with varying radius and height (see Supporting Information: Complex topology). These topologies serve as the basis for fabricating engineered synthetic skeins. Two different hidden length ratios: λ_max _ = 101 and λ_max _ = 787, where λ_max _ = *L*_*f*_/*H*, and *L*_*f*_ is the total arc length of the helical path, *d*_*f*_ ⩽ 103 µm and 11 µm, respectively, and *D*_*o*_ = *H* = 6 mm is the outer dimension of the topology, are considered. The smaller pitch produces a more compact initial configuration, resulting in a higher hidden length ratio (λ ⩽ 787 compared to λ ⩽ 101) when uncoiled. Our objective is to determine the tradeoffs between fiber diameter *d*_*f*_ and material property ε_break_, to meet the coil‐uncoil requirements for this topology.

The radius of curvature distribution ℜ(*s*), where *s* is the arc length coordinate, is analyzed to understand the geometric characteristics of the synthetic thread topology. The probability density is constructed using Kernel Density Estimation (KDE) to capture the likelihood of different radius of curvature values occurring along the thread's length as $P\left(ℜ\right)=\frac{1}{Nh\sqrt{2\pi }}\sum_{i=1}^{N}\exp\left(-\frac{{\left(ℜ-{ℜ}_{i}\right)}^{2}}{2h^{2}}\right)$where *h* = 0.0372 mm is the smoothing bandwidth that controls the degree of smoothing, *N* = 1000 is the total number of radius of curvature data points used to construct the distribution, and $\int_{0}^{L_{f}}p\left(ℜ\right)\;dℜ=1$. The probability density, shown in Figure 3e, highlights values of radius of curvature, ℜ, most frequently observed (mode(s) of the KDE distribution). Higher probability density corresponds to values of ℜ that are more prevalent in the topology, indicating thread sections with consistent or repeated curvature. Increasing λ shifts the coil geometry toward tighter packing, with more segments exhibiting lower ℜ. With this configuration, higher λ necessitates more extreme bending, increasing the mechanical demands on fiber flexibility.

We can relate the infill void ratio, 2ℜ_min _/*D*_*o*_, and the feature size ratio, *D*_*o*_/*d*_*f*_, using equations obtained from the nested cylindrical topology as illustrated in Figure 3f with vertical lines representing the range of void ratios corresponding to the most frequently observed curvature. This reveals that *D*_*o*_/*d*_*f*_ is relaxed for λ_max _ = 101, with values close to 10, approximately three times less than λ_max _ = 787. Thus, to achieve a sevenfold higher λ with the same *D*_*o*_, the thread diameter *d*_*f*_ must be at least three times smaller.

The curvature distributions allow us to estimate the strain associated with uncoiling for these topologies. Both topologies share a mean radius of curvature, ℜ = 0.86 mm, thus the associated mean strain is proportional to the thread diameter *d*_*f*_ and, interestingly, less for skein topology with a higher λ (as one needs to use smaller *d*_*f*_ to achieve the required λ). In this case, a fiber diameter *d*_*f*_ = 10 µm requires a material with ϵ_break_ ⩾ 5.8%. Larger fiber diameters *d*_*f*_ are acceptable and can even simplify the manufacturing processes. However, this comes with a trade‐off as larger *d*_*f*_ increases the strain demands during uncoiling, necessitating materials with higher ϵ_break_. As fabrication becomes more accessible at larger scales, material selection becomes more restrictive. Moreover, complete straightening of the threads after unraveling is not always necessary, and observation of natural slime in Figure 1 reveals remnant curvature in the unraveled threads. Quantitatively, the fully uncoiled configuration corresponds to the geometric limit where there is no slack (discussed in section 5.2). A material with a lower ϵ_break_ can also be selected if the fiber is not required to fully unravel into a straight configuration, i.e., from Equation 2, ℜ_*f*_ being finite rather than infinite will relax the requirement on ϵ_break_.

In summary, the strain requirements for uncoiling are closely linked to fiber diameter *d*_*f*_ and hidden length ratio λ, with smaller fiber diameters, *d*_*f*_, reducing strain demands but presenting greater manufacturing challenges. While larger *d*_*f*_ values simplify fabrication, they require materials with higher strain‐to‐break limits to enable full uncoiling. These trade‐offs must also consider other functional requirements, leading to the next key question: how can fibers be held in a coiled or uncoiled state while resisting the release of their stored bending energy?

## 3 Principles of Holding Elastic Energy in A Non‐Equilibrium Condition

**Figure 4:**
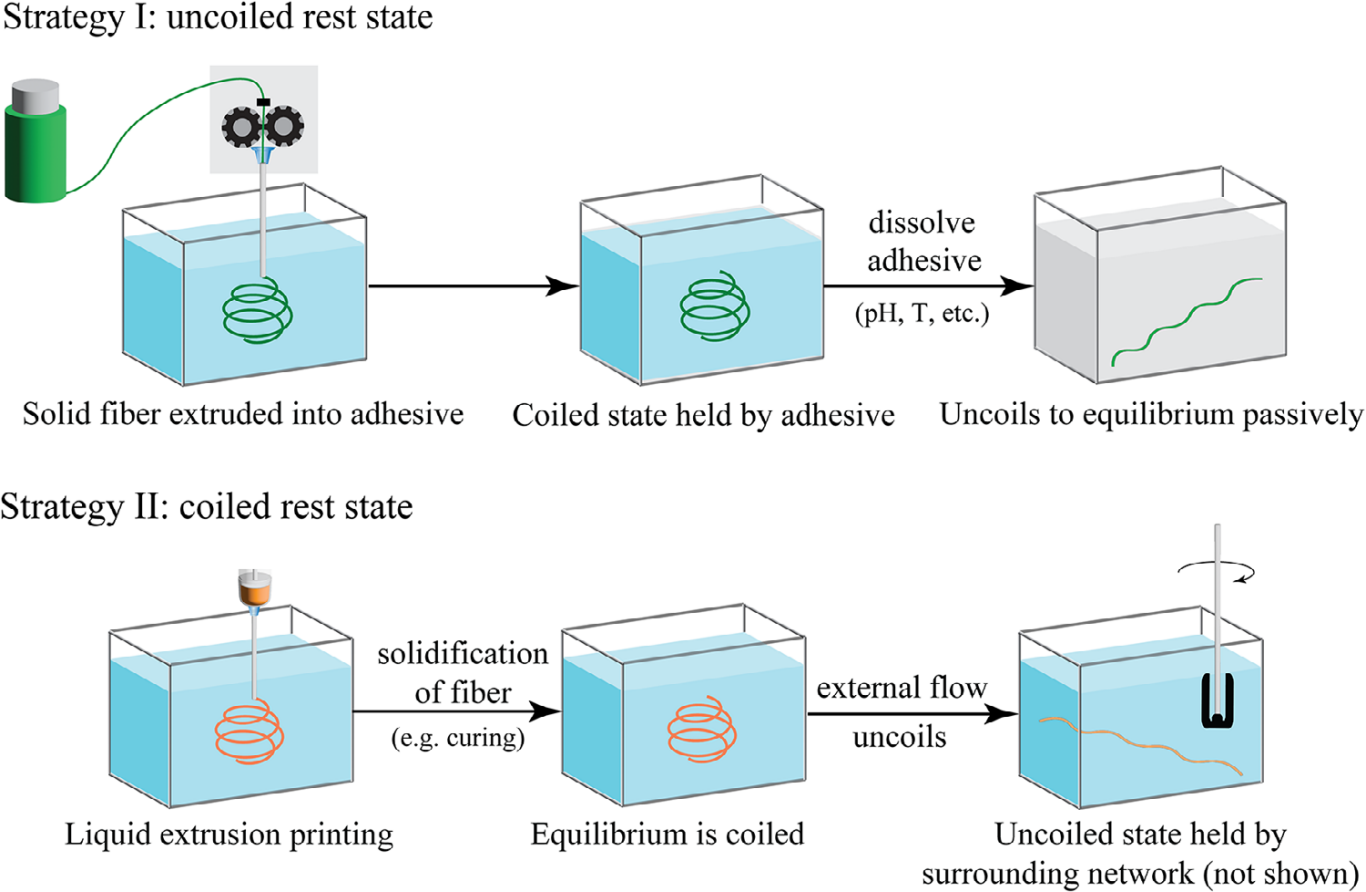
Two different strategies are proposed to achieve the goal of a targeted hidden length ratio, each based on a distinct equilibrium condition for the thread. In Strategy I, solidification occurs before extrusion, with the equilibrium state being the extended configuration (uncoiled rest state). The thread stores elastic energy in the coiled state with the assistance of adhesives. Upon dissolving the adhesive, the thread returns to its extended form. In Strategy II, solidification occurs after extrusion, where the equilibrium state is the coiled configuration (coiled rest state). External flow forces are required to stretch the fiber into the extended state and hold it in a non‐equilibrium state that overcomes the thread's natural tendency to remain coiled.

Elastic restoring forces are important for deployable fiber networks, as fibers inherently seek to return to their equilibrium configurations. In the worst case, assuming fully elastic behavior and negligible plastic deformation, both coiled and uncoiled states may contain significant stored elastic stresses. This behavior, inherent to natural skeins, becomes a critical design challenge for synthetic skeins. Synthetic skeins can be designed and fabricated with either (i) an uncoiled rest state, where stored elastic energy drives uncoiling and maintains the extended configuration, or (ii) a coiled rest state, where the fiber remains coiled until flow initiates unraveling, requiring external constraint to maintain a non‐equilibrium extended configuration (**Figure** **4**). Both strategies rely on maintaining an elastic fiber in a non‐equilibrium state, e.g. achieved by adhesives/encapsulants/yield stress fluids that mimic the role of the natural skein adhesive, where the yield strength of the surrounding material serves as the key metric for providing restraining forces.

The design space involves key trade‐offs: larger *d*_*f*_ will ease fabrication but may require extremely large yield strength σ_*ys*_ of the surrounding medium to hold non‐equilibrium conditions, while smaller *d*_*f*_, though harder to manufacture, is easier to maintain in a non‐equilibrium state with lower σ_*ys*_. Such trade‐offs can expand the design framework beyond biomimicry, enabling the rational selection of fiber properties to balance manufacturing constraints and network deployment.

Here, we analytically model how the yield strength σ_*ys*_ of the surrounding relates to the fiber diameter *d*_*f*_, modulus *E*_*f*_, and radius of curvature ℜ to hold an elastic fiber in a non‐equilibrium state (Section 3.1), followed by experimental tests of the theory (Section 3.2).

### 3.1 Results: Required Yield Strength to Hold the Non‐Equilibrium State

Strategy I (Figure 4) considers solidification before extrusion (extended rest state). In this case, the adhesive/encapsulant/yield stress fluid holds the fiber in a coiled state, e.g., to hold a certain curvature of the thread, resisting the elastic stiffness of the fiber, which would otherwise spring back to a straight configuration. The stored elastic energy can be responsible for passive unraveling if the thread has sufficient elasticity. Manufacturing techniques to extrude solid fibers into a yield stress medium in complicated trajectories are restricted to larger thread diameters.^[^ ^30^ ^]^ However, this method takes advantage of a passive unraveling process, which occurs automatically once the medium dissolves, allowing the stored elastic energy to return the thread to its original, straight configuration. The proof‐of‐principle is demonstrated in Figure S2 (Supporting Information), where a soft elastic fiber is confined in a Carbopol yield‐stress gel (σ_*y*_ = 113 Pa) and was maintained in a non‐equilibrium coiled state. Upon gel dissolution in NaCl solution, it recoils to its equilibrium configuration.

Strategy II (Figure 4) involves solidification occurring after extrusion, resulting in a coiled rest state. This approach aligns with polymer processing and additive manufacturing processes,^[^ ^25^, ^26^, ^27^, ^31^, ^32^ ^]^ where the intrinsic elasticity of the fiber does not induce spontaneous unraveling, and requires external forces to unravel. If the elastic stress stored in the extended state is not adequately managed, the threads can recoil and collapse without a surrounding network or yield stress fluid.

In both strategies, the required yield strength of the surrounding medium will be a function of thread properties and geometry, i.e., stiffness, cross‐section, length, and curvature. As a base case, assuming linear elastic deformation of a filament with a circular cross section, we can write

(4) $$\sigma_{ys}=f\left(E_{f},d_{f},ℜ,L_{f}\right)$$

where *E*_*f*_ is the elastic modulus of the fiber, *d*_*f*_ is the diameter, ℜ is the local radius of curvature, and *L*_*f*_ is the total length of the thread. This simplified model neglects effects such as viscoelasticity of the medium, material anisotropy, and geometric imperfections, which may become important under specific conditions. Using the Buckingham Π theorem on Equation 4 results in a reduced relationship between dimensionless groups that we can write as

(5) $$\frac{\sigma_{ys}}{E_{f}}=\phi \left(\frac{d_{f}}{ℜ},\frac{d_{f}}{L_{f}}\right).$$

To get insight into the functional dependence of σ_*ys*_/*E*_*f*_ on geometric parameters, we consider the case of a fiber placed in a yield stress fluid, where the yield strength σ_*ys*_ corresponds to the material yield stress σ_*y*_. A similar framework also applies to adhesive systems, where the resisting force is governed by the adhesive strength rather than a bulk yield stress. An elastic fiber with a certain curvature embedded within an unyielded yield stress fluid is statically indeterminate because the exact distribution of resisting stress from the surrounding medium is unknown. However, we can gain insight by considering limiting cases.

**Figure 5:**
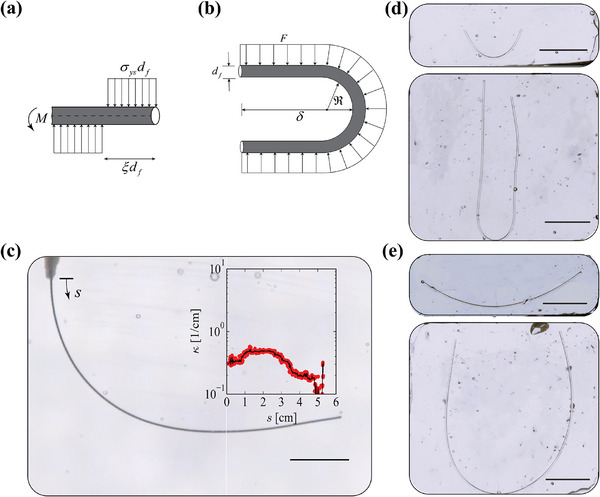
Holding elastic bending of fibers within a yield stress fluid. a) local moment balance, b) non‐local moment balance for a fiber held in a U‐shape. c) Experiments with thread *E*_*f*_ ≈ 2 GPa, *d*_*f*_ = 0.2 mm and σ_*y*_ = 113 Pa showing the equilibrium minimum radius of curvature in a Carbopol microgel yield stress fluid (Carbopol 980, 1 wt%, pH ≈ 7). The inset shows the curvature distribution along the arc length of the thread. d) Minimum stable radius of curvature for threads of two different lengths with *E*_*f*_ ≈ 40 MPa, *d*_*f*_ = 0.47 mm and σ_*y*_ = 69.6 Pa in Carbopol microgel (0.5 wt%, pH ≈ 7). e) Minimum stable radius of curvature for threads of two different lengths with *E*_*f*_ ≈ 2 GPa, *d*_*f*_ = 0.5mm and σ_*y*_ = 69.6 Pa. All scale bars are 1 cm.

**Figure** **5**a considers a worst‐case setting where the gel resists only over a small region near the free end of the filament. For example, the yield stress of the gel can provide a resisting resultant torque that opposes the internal bending moment generated by the fiber's curvature. Considering a local moment balance, using classical beam theory, we can estimate the minimum resisting surrounding stress required to sustain this bending. The local internal bending moment in the fiber is *M* = *E*_*f*_ *I*/ℜ, where $I=\pi d_{f}^{4}/64$ is the second moment of area and ℜ is the local radius of curvature. We assume the yield stress fluid applies a local force couple over a distance of ξ*d*_*f*_, where ξ is an order one dimensionless coefficient acknowledging the unknown length of the distributed load. The total resisting force per unit length exerted by the surrounding yield stress fluid is then approximated as σ_*ys*_ · *d*_*f*_, generating a resultant moment *M* = σ_*ys*_ *d*_*f*_ · (ξ*d*_*f*_)^2^. The internal moment in the fiber may depend on the distance away from the free end, with a characteristic moment located at the small distance 2ξ*d*_*f*_ from the free end. At that location, balancing the moments and solving for the required yield strength σ_*ys*_ gives

(6) $$\sigma_{ys}=\frac{\pi }{64}\frac{E_{f}d_{f}}{ℜ}\frac{1}{\xi^{2}}.$$

This expression highlights key trends: tighter coils (smaller ℜ) require higher yield stress, stiffer fibers (larger *E*_*f*_) and thicker fibers (larger *d*_*f*_) are more difficult to bend. This form is consistent with the classical internal bending stress expression and provides a useful scaling framework for estimating the yield strength required to maintain fiber curvature within a yield stress fluid. Experimental observations with actual threads (Figure 5c) reveal that Equation 6 may be reasonable for a free end of a thread (e.g., minimal curvature near the free ends in Figure 5c), but does not predict the tight curvature in the interior regions of the thread, suggesting that it indeed represents a worst‐case scenario. We hypothesize that the nonlocal effects must be considered to understand how moments are generated to maintain a bend in the long filament.

Figure 5b considers nonlocal effects where the resultant force on the thread is distributed over a certain length scale δ. Approximating the force as uniformly distributed over the length δ and applying a moment balance yields (see Supporting Information for details)

(7) $$\begin{array}{cc} \sigma_{ys} & =\frac{\pi }{32}\cdot \frac{E_{f}d_{f}^{3}}{{ℜ}^{3}}\;\text{for}\;\delta =ℜ, \end{array}$$

(8) $$\begin{array}{cc} \sigma_{ys} & =\frac{\pi }{8}\cdot \frac{E_{f}d_{f}^{3}}{ℜL_{f}^{2}}\;\text{for}\;\delta =L_{f}/2. \end{array}$$

Equations 7 and 8 give the best‐case scenario, i.e., the lowest yield strength required. As an example calculation, for a native hagfish thread of *E*_*f*_ = 6 MPa, *d*_*f*_ = 3 µm, ℜ = 50 µm and *L*_*f*_ = 100 µm, Equations (6), (7), and 8 result in predicted required yield strengths of 180 kPa, 130 kPa, and 1.2 kPa, respectively. Increasing the diameter to *d*_*f*_ = 30 µm, e.g., due to manufacturing constraints, increases σ_*ys*_ in each case. Thus, increasing *d*_*f*_ will constrain ℜ for the same yield strength of the surrounding material.

### 3.2 Results: Experiments on Holding Coiled Fibers Using Yield Stress Fluids

Simple experiments were conducted where a thread is placed in a yield stress fluid as shown in Figure 5c–e to validate the modeling. We used 1 wt% (Figure 5c) and 0.5 wt% (Figure 5d,e) Carbopol 980 microgel systems as model yield stress mediums. Dynamic yield stress (σ_*y*_) of Carbopol microgel was obtained by fitting steady shear rheometry data to a Herschel‐Bulkley equation of the form^[^ ^33^ ^]^ $\sigma =\sigma_{y}\left(1+{\left(\dot{\gamma }/{\dot{\gamma }}_{crit}\right)}^{n}\right)$(Figure S3, Supporting Information, adapted from Hossain et al.^[^ ^28^ ^]^). Commercially available polyamide/polyurethane threads with diameters of *d*_*f*_ = 0.2, 0.47, 0.5  mm were carefully embedded in the fluid to replicate the schematic shown in Figure 5b, initially at high curvature, which relaxed to a stable (lower) curvature when external force was released. The Young's modulus of the fibers was measured using a torsional fixture on an ARES‐G2 rheometer in axial mode (Figure S3, Supporting Information), which gives *E*_*f*_ ≈ 40 MPa (polyurethane, Figure 5d), and ≈2~GPa (polyamide, Figure 5c,e). For the configuration in Figure 5c, a thread segment (*E*_*f*_ ≈ 2 GPa) approximately 5.3 cm in length was placed in the gel such that one end was fixed while the other end remained free to move (Figure S4, Supporting Information). The curvature distribution of the thread along the arc length is shown in the inset of Figure 5c with an average radius of curvature at the middle section ℜ = 2.2 cm. Equation 8 predicts the minimum required yield strength σ_*ys*_ ∼ 106 Pa for this setting, which aligns with the fact that the yield stress of the microgel in Figure 5c is 113 Pa.

Figure 5d and e present the resulting radii of curvature for fibers with a diameter of *d*_*f*_ ≈ 0.5 mm embedded in Carbopol microgel with a yield stress of σ_*y*_ = 69.6 Pa. The non‐equilibrium radius of curvature, ℜ, plays a central role in design, as it dictates the non‐equilibrium shape for a given *d*_*f*_, *L*_*f*_, and σ_*adh*_. For the softer fiber (*E*_*f*_ ≈ 40 MPa) shown in Figure 5d, inverting Equation 7 and 8 predicts equilibrium radii of curvature of approximately ℜ = 19 mm and ℜ = 5.8 mm for thread lengths of *L*_*f*_ = 23 mm and *L*_*f*_ = 70 mm, respectively. The experimentally observed radii of curvature, ℜ = 6 mm and ℜ = 4 mm, are in reasonable agreement with these predictions.

For the stiffer fiber (*E*_*f*_ ≈ 2 GPa) shown in Figure 5e, the model of Equations 7 and 8 predict larger stable radii of curvature of approximately ℜ = 66 mm and 120 mm for thread lengths of *L*_*f*_ = 41 mm and 100 mm, respectively. However, the experimentally observed curvatures are smaller, with ℜ = 30 mm and 15 mm, respectively. Notably, the equilibrium configuration of this fiber within the gel differs substantially from the idealized model geometry depicted in Figure 5b. This discrepancy indicates a regime where more advanced modeling approaches are needed to accurately capture the complex, nonlocal interactions between elastic threads and yield‐stress fluids.

Our current model assumes linear elastic behavior, neglecting potential nonlinear elasticity or plastic deformation of the fiber, particularly at high strains. Additionally, the filament geometry is simplified to a uniform U‐shape, whereas real threads may display complex, multiscale curvature and non‐uniform packing. Furthermore, we assume a simple yield‐stress fluid model, neglecting any viscoelastic deformation before yielding. We also note that material‐specific effects, such as the moisture sensitivity of polyamide fibers, can lead to small changes in modulus and diameter over long immersion times,^[^ ^34^ ^]^ although such swelling effects are likely negligible, especially under the short (∼5 min) immersion times used in our experiments. These complexities, while beyond the scope of the present model, may be required for accurate performance prediction and could be addressed in future work. Nevertheless, the design principles revealed by the analytical model, and Equation 8 specifically, provide critical guidance for material selection and sensitivity to geometric parameters.

In summary, the required σ_*ys*_ scales directly with *E*_*f*_ and *d*_*f*_, and inversely with ℜ. Consequently, smaller and softer fibers demand lower yield strengths of the surrounding, whereas tighter coils (smaller ℜ) require higher yield strength to retain the elastic energy of the fiber in its non‐equilibrium state. In this analysis, we have focused exclusively on elastic fibers, ensuring that the coil‐uncoil transition does not induce plastic deformation. Introducing plasticity would reduce the yield strength requirement, as the fiber would no longer seek to recover its original shape; however, yield stress fluids may still be necessary to counteract other effects, such as gravitational sagging of the skein in both strategies^[^ ^35^, ^36^ ^]^ (see Supporting Information for details). Importantly, our results confirm the feasibility of using yield‐stress fluids/adhesives to maintain manufacturable *d*_*f*_ in a non‐equilibrium configuration. Notably, larger *d*_*f*_, which is advantageous from a manufacturing perspective, remains feasible when combined with higher ℜ, provided that the yield strength is appropriately selected.

## 4 Principles of Fluid‐Mediated Unraveling of Coiled Elastic Fibers

Coiled hagfish threads are released and unravel very quickly in fluid flow (a few hundred milliseconds); the short time is important for their functionality. Chaudhary et al.^[^ ^10^ ^]^ demonstrated theoretically that viscous hydrodynamics can dominate this process, enabling unraveling in less than 1 s, comparable to the natural slime formation timescale. Crucially, unraveling occurs only if the viscous drag exceeds the skein's peeling resistance, characterized by the peeling number ℘, with the unraveling time subsequently determined by the flow strength.^[^ ^10^, ^15^ ^]^ However, it is essential to recognize that natural skeins likely involve additional complexities and unknown factors, such as biological adhesion mechanisms and chemistry, which may further influence unraveling dynamics. Although the biological scenario of unraveling is complicated by the surrounding seawater, which transmits forces through the mucus matrix,^[^ ^15^ ^]^ here the yield‐stress medium serves as a simplified analogue, either mediating stress transfer under flow or acting as a dissolvable capsule under quiescent conditions.

We consider a range of possible synthetic approaches for unraveling, e.g., with or without passive uncoiling (Figure 4). If an adhesive/yield stress fluid maintains the fiber in a coiled state by resisting its elastic restoring force (strategy I), then rapid dissolution of the medium, e.g., as can be triggered by temperature or pH changes for yield stress fluids, as in Pluronic F127^[^ ^37^ ^]^ or Carbopol,^[^ ^38^ ^]^ could release the stored elastic energy to drive passive unraveling. On the other hand, threads can unravel due to external flow as the surrounding fluid (or yield‐stress gel) transmits hydrodynamic forces from the flow to the fiber (strategy II).

Two key aspects must be considered: the extent of unraveling and the unraveling timescale. Smaller *d*_*f*_ may lead to longer unraveling times due to lower stored elastic energy (strategy I) and reduced viscous traction (strategy II). In strategy I, unraveling proceeds upon adhesive/yield stress fluid dissolution; in contrast, strategy II depends on whether fluid forces can overcome the bending resistance of the fiber.

Here, we establish scaling relations that link the thread properties (*d*_*f*_, *E*_*f*_, *L_f_*) to the fluid properties (considering viscoplastic fluid with yield stress σ_*y*_) and the hydrodynamic flow conditions ($\dot{\varepsilon },\dot{\gamma }$) necessary for unraveling with strategy II. We show that unraveling can occur for fibers with a higher modulus and larger diameter compared to hagfish threads, given sufficient flow strength. High‐fidelity modeling, such as solving the full governing equations of fluid flow with elastic fiber and surrounding medium interactions, is not considered here, as its necessity is not yet clear. We also gain insights into the unraveling time *t*_unravel_ using a non‐Brownian bead‐spring model, to understand the hydrodynamic flows (strain rates $\dot{\varepsilon },\dot{\gamma }$) required for unraveling a hidden length ratio λ.

### 4.1 Results: Unraveling Criteria for Elastic Fibers in a Viscoplastic Medium

The unraveling of natural skeins can be driven by viscous drag forces, which must overcome the peeling resistance of the fibers. The dimensionless ratio of viscous drag to peeling resistance, denoted as the Peeling number ℘ = *F*_*D*_/*F*_*P*_, dictates the onset and speed of unraveling.^[^ ^10^ ^]^ When ℘ is large, unraveling occurs rapidly, approaching a kinematic limit where the local flow directly advects threads.

While the Peeling number captures the balance of drag and adhesion, an alternative but related perspective considers the fiber's bending resistance as the primary barrier to deformation, especially in synthetic systems or non‐adhesive media. For a fiber in a Newtonian fluid, the local balance between viscous forcing and elastic restoring forces—modeled using slender body theory (SBT) or bead‐spring representations—leads to a dimensionless parameter^[^ ^39^ ^]^

(9) $$\bar{\eta }=\frac{8\pi \eta_{s}\dot{\gamma }L_{f}^{4}}{E_{f}Ic}$$

where *c* = −ln [*e*(*d*_*f*_/(2*L*_*f*_))^2^], *L*_*f*_ is the length of fiber, η_*s*_ is the solvent viscosity, and $\dot{\gamma }$ is the flow strength. When $\bar{\eta }\gg 1$, the fluid flow overcomes the bending resistance of the fiber. This scaling can be inverted to yield a critical shear rate criterion, where unraveling occurs when the applied shear rate exceeds a threshold value determined by fiber stiffness and geometry: ${\dot{\gamma }}_{\text{crit}}\gg E_{f}d^{4}/\left(\eta_{s}L_{f}^{4}\right)$.

In non‐Newtonian environments such as yield‐stress fluids, unraveling requires that the applied flow stress first exceeds the yield stress of the surrounding medium to allow deformation, and then surpasses the fiber bending resistance to initiate uncoiling. This sets a minimum required flow strength governed by both the yield stress of the fluid and the elastic bending stiffness of the fiber, characterized by its modulus *E*_*f*_ and geometric second moment of area *I*.

**Figure 6:**
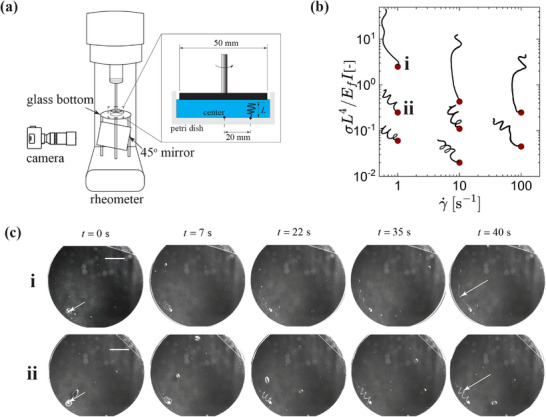
Fluid‐mediated unraveling of coiled elastic fibers. a) Experimental setup: optical access on a rotational rheometer with a custom‐made glass bottom plate attached with 45^o^ angle mirror. Coiled fibers were printed in a petri dish containing yield stress fluids at a 20 mm distance from the center. One end of the fiber was rooted at the petri dish substrate during the printing process. A 50 mm diameter parallel plate was used to impose controlled flow to unravel the coiled fibers. b) Regime map of conditions (symbols) with Elasto‐Plastic number EP=σ*L* ^4^/(*E*_*f*_ *I*) and shear rate, along with the final experimental profile of the fiber (black line extending from anchor point). c) Time‐lapse images showing fluid‐mediated unraveling of coiled fibers with two different thread diameters: (i) 140 µm and (ii) 250 µm. Scale bar is 10 mm.

To investigate the unraveling criteria for synthetic skeins, we developed an experimental setup using a rheometer, as shown in **Figure** **6**a. Coiled springs were 3D‐printed (using embedded 3D printing by solvent exchange^[^ ^27^ ^]^) from Styrene Ethylene Butylene Styrene (SEBS) Block Copolymer with *E*_*f*_ = 23 MPa,^[^ ^27^ ^]^ each with a height and coil diameter of *D*_*o*_ = 3 mm and comprising three turns, with one end anchored to a Petri dish during printing with a range of fiber diameters, *d*_*f*_ = 140 − 540 µm (details of the printing process, characterization of the ink and bath medium is provided in Experimental Section; Figures S5 and S6, Supporting Information). After printing the coiled springs in a yield stress fluid, the entire Petri dish was placed in a rotational rheometer equipped with a custom‐made glass bottom plate. A 45° mirror was positioned beneath the setup, with a camera directed at the mirror to capture the rheometer from below. A 50 mm parallel plate was attached to the top, applying a constant rotation rate to induce the unraveling of the coiled spring. Figure 6c presents two examples: one with a fiber diameter of 140 µm (i) and another with 250 µm (ii), both subjected to a shear rate (at the plate edge) of 1 s^−1^ in a yield stress fluid with σ_*y*_ = 6.7 Pa. The fiber with the larger diameter (greater bending resistance) did not significantly deform in the flow, unlike the thinner fiber, which successfully deployed (Movie S1).

Motivated by these observations, we seek to capture key insights by modeling the fiber as a cantilevered beam subjected to distributed fluid stresses. While the actual deployment of coiled fibers involves unsteady flow, complex geometries, and localized deformations, a simplified model can still provide useful design guidelines. In this model, the tip deflection under a uniformly distributed load is given by Δ = *wL* ^4^/(8*E*_*f*_ *I)* where *w* is the force per unit length imposed by the surrounding fluid, *L* is the coiled fiber length, *E*_*f*_ is the elastic modulus, and *I* is the second moment of area of the cross section(Figure 6a).^[^ ^40^ ^]^ In parallel plate flow, where the stress remains uniform along the height, the load on the beam can be expressed as *w* ≈ σ*d*_*f*_, where σ is the total stress exerted by the fluid. Substituting into the deflection equation,

(10) $$\frac{\Delta }{d_{f}}=\frac{1}{8}\frac{\sigma L^{4}}{E_{f}I}.$$

The ratio Δ/*d*_*f*_ quantifies the beam deformation relative to the fiber diameter. When Δ/*d*_*f*_ ≫ 1, the deformation is significant, indicating a strong tendency for unraveling. For a viscoplastic fluid, the stress σ, as a function of shear rate, is determined using the Herschel–Bulkley (HB) model, $\sigma =\sigma_{y}\left(1+{\left(\dot{\gamma }/{\dot{\gamma }}_{\text{crit}}\right)}^{n}\right)$. Thus, for viscoplastic fluid, this analysis reveals a dimensionless parameter that we call the Elasto‐Plastic (EP) number, given by:

(11) $$\text{EP}=\frac{\sigma L^{4}}{E_{f}I}.$$

This scaling is consistent with Equation 9, when $\sigma =\eta_{s}\dot{\gamma }$ represents the flow‐induced Newtonian stress, corresponding to the same scaling.

To rationalize the observed unraveling behavior in synthetic systems, we quantify the EP number across a range of shear rates and fiber diameters, aiming to identify the critical threshold required for successful unraveling. We do observe small amounts of recoil after flow cessation, which we attribute to the difference between the applied flow stress and the yield stress of the surrounding medium. However, the unraveled fibers do not completely recoil back; rather, they remain in their stretched state once the flow is stopped (Figure S7, Supporting Information). Figure 6b presents results for fibers of different diameters, showing the corresponding EP number as a function of the applied shear rate. These values are calculated using a yield‐stress fluid with σ_*y*_ = 6.7 Pa, ${\dot{\gamma }}_{\text{crit}}=2.86$ s^−1^, and *n* = 0.47. Overlaid on the plot are the final configurations of the fibers at each condition. While a general trend of increased unraveling with higher EP numbers is observed, the transition is more nuanced than a strict threshold. At higher shear rates, fibers with relatively low EP values also unravel, suggesting that secondary effects may contribute to the response. We observe unraveling occurs for free‐ended coiled fibers (Figure S8, Supporting Information), which can also be rationalized by the EP number (≈0.6). Furthermore, when multiple coiled geometries exist in non‐dilute conditions, the interactions may become important in the unraveling process. For example, direct entanglements between fibers could rapidly accelerate extension and alter the unraveling dynamics compared to the single‐thread case. Nonetheless, the results in Figure 6c reveal predictive insight from the EP number, and show how the unraveling of a coiled thread is possible even when its diameter is 70 times larger than that of hagfish threads, provided that sufficient stress is applied.

In summary, synthetic skeins can successfully unravel under fluid‐mediated conditions even with significantly higher *E*_*f*_ or *d*_*f*_ compared to natural systems, as long as the applied fluid stresses exceed a critical threshold. While this simple trend should be expected, our modeling and experimental results support the specific scaling prediction that the critical stress scales as $\sigma_{\text{crit}}∼E_{f}d_{f}^{4}/L^{4}$, indicating a strong sensitivity to fiber diameter. Therefore, this framework broadens the design space by providing predictive guidelines for selecting material and geometric properties to achieve successful unraveling in synthetic systems.

### 4.2 Results: Unraveling Timescale of Elastic Coiled Fibers in Viscous Flow

To design synthetic skeins that unravel rapidly in flow, it is important to understand how unraveling time depends on both material properties and flow conditions. Specifically, we seek to answer what flow strength is required to achieve rapid deployment, and how fiber properties influence this threshold. Chaudhary et al.^[^ ^10^ ^]^ showed that when ℘ is large, unraveling occurs rapidly, approaching a kinematic limit where the local flow directly advects threads. For a characteristic velocity *U*, the deployment time in simple shear flows scales as *t*_unravel_ ∼ *L*_max_/*U*, while in extensional flows with strain rate $\dot{\varepsilon }$, it scales as $t_{\text{unravel}}∼{\dot{\varepsilon }}^{-1}\ln\left(\lambda \right)$, where λ = *L*/*L*_*i*_ is the hidden length ratio.^[^ ^10^ ^]^

When synthetic skeins adopt a coiled rest state (strategy II), unraveling requires external forces, and the unraveling time may depend on a combination of fiber, fluid, and flow properties, captured by the functional relationship $t_{\text{unravel}}=f\left(d_{f},L_{f},\eta_{s},E_{f},\dot{\gamma },\dot{\varepsilon }\right)$ where *d*_*f*_ is the fiber diameter, *L*_*f*_ is the thread length, η_*s*_ is the fluid viscosity, *E*_*f*_ is the fiber modulus, and $\dot{\gamma }$, $\dot{\varepsilon }$ represent shear and extensional strain rates, respectively. This scaling neglects fiber–fiber interactions, assumes a low Reynolds number, and negligible inertia. However, it captures the leading‐order sensitivity of unraveling dynamics to fiber geometry, material stiffness, and flow strength, offering a useful framework for understanding design trade‐offs in synthetic skeins.

To obtain first‐order predictions, we use bead–spring models assuming neglegible inertia and idealized deformation, allowing us to identify the controlling parameters and provide lower‐bound estimates for the required flow strength. Two limiting cases are considered: (i) extensional flow that pulls the skein apart, and (ii) simple shear flow that peels the skein from a surface (Figure S9, Supporting Information).

In extensional flow, the unraveling time follows $t_{\text{unravel}}={\dot{\varepsilon }}^{-1}\ln\left(L_{f}/L_{i}\right)$ where λ = *L*_*f*_/*L*_*i*_ is the hidden length ratio (same scaling as,^[^ ^10^ ^]^ see SI for details). Achieving λ = 1000 within 0.4 s (as in hagfish) requires $\dot{\varepsilon }=17\;s^{-1}$. This corresponds to a velocity gradient of just 2.6 mm/s across a 150 µm skein. For a given flow field, a synthetic skein with a larger *D*_*o*_ experiences a smaller local extension per unit length; thus, a greater velocity gradient is needed to impose the same effective extensional strain rate.

Adding thread elasticity would slow down the unraveling. Considering the elasticity of the threads (a linear elastic spring with spring constant *H* slows the unraveling), the unraveling time becomes $t_{\text{unravel}}∼\ln\lambda /\left(\dot{\varepsilon }-H/(3\pi \eta_{s}a\right))$ where *a* is the diameter of the bead (see Supporting Information for details). Unraveling time will follow the kinematic limit if $H/(3\pi \eta_{s}a\dot{\varepsilon })\ll 1$, i.e., when fluid viscosity and strain rate dominate over fiber elasticity. Assuming the spring constant scales as $H∼E_{f}d_{f}^{2}/L$, this relationship shows that increasing the fiber modulus *E*_*f*_ or diameter *d*_*f*_ increases elastic resistance and thus slows down unraveling.

In shear flow, unraveling time scales as $t_{\text{unravel}}=\left(L_{f}-L_{i}\right)/(\dot{\gamma }y_{0})$ where *y*_0_ is the distance from the skein to the surface (same scaling as,^[^ ^10^ ^]^ see Supporting Information for details). For λ = 1000, achieving 0.4 s unraveling requires $\dot{\gamma }=2500\;s^{-1}$, nearly two orders of magnitude higher than for extensional flows.

These results establish practical flow thresholds for synthetic skein unraveling dynamics. Specifically, unraveling times are governed by ratios such as ($H/(3\pi \eta_{s}a\dot{\varepsilon })$) and the hidden length ratio λ = *L*_*f*_/*L*_*i*_. Extensional flows are far more efficient for rapid unraveling, and fiber elasticity can be tolerated when the surrounding viscosity is high.

## 5 Principles and Requirements for A Soft Network

**Figure 7:**
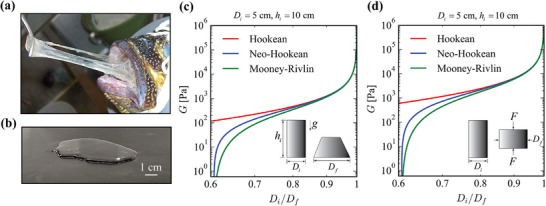
Principles and requirements for a soft network similar to hagfish slime. a) Hagfish slime clogging the gills of a fish, reused with permission from Lim et al. (2006).^[^ ^41^ ^]^ b) Hagfish slime undergoing large deformation due to gravitational forcing, forming a puddle. c) Required shear modulus to enable radial deformation from *D*_*i*_ to *D*_*f*_ due to gravitational forcing. d) Required shear modulus as a function of the diameter ratio, subjected to a 10 N compressive force. In both (c) and (d), the parameters are *h*_*i*_ =10 cm and *D*_*i*_=5 cm, ρ = 1000 kg/m^3^, ν = 0.5, and *C*_10_/*C*_01_ = 3 for the Mooney‐Rivlin model. All three models are identical in the limit of small strain.

A final requirement demanding sufficiently soft and thin fibers is that the hagfish slime network is remarkably soft, conforming to its surroundings, which is essential to its function in clogging predator gills (**Figure** **7**a). Its extreme deformability allows it to conform to complex surroundings, enhancing its defensive efficacy.^[^ ^41^ ^]^ The precise criteria for achieving the necessary deformability, both at the network scale and at the level of individual threads, remain unclear; however, it is important to determine the required *d*_*f*_, *E*_*f*_, e.g., can thicker *d*_*f*_ fibers still form a sufficiently soft network?

As a functional requirement of a soft network, we considered the ability of the material to conform to its surroundings, akin to hagfish slime's capacity for large deformations, as shown in Figure 7b. Although hagfish slime behaves as a soft solid with a shear modulus of *G* ≈ 0.02 Pa, it undergoes significant deformation, often resembling a puddle of liquid, demonstrating its extreme network softness. To understand the necessary conditions for achieving similar behavior in synthetic networks, we identified two key properties: (1) the network modulus *G* required to enable large deformations similar to hagfish slime, and (2) the fiber modulus *E*_*f*_ and diameter *d*_*f*_ needed to form such a soft network.

Here, we develop an analytical model to explore the essential trade‐offs between single‐thread properties and resulting network mechanics, aiming to identify the conditions required for a soft, deformable fibrous network. Our goal is not to fully replicate the complexity of hagfish slime, but rather to extract governing relationships and sensitivity trends that guide design. Using a coarse‐grained mesoscopic framework,^[^ ^18^ ^]^ we define quantitative targets for modulus and deformability based on the requirement to deform significantly under gravitational and compressive stress. While hagfish slime sets an extreme benchmark for softness, our analysis reveals that synthetic analogs can achieve functionally similar behavior without matching this extremity, and a broader design space is accessible by using larger *d*_*f*_ or higher *E*_*f*_ fibers.

### 5.1 Results: Continuum‐Level Material Property Requirements

Here, we quantitatively derive softness requirements for a whole‐slime network to deform to its surroundings using large deformation models subjected to both body and compressive boundary forces. As an instructive case, we considered a cylindrical mass (inset of Figure 7c) that deforms under the action of gravity or fixed compressive force (inset of Figure 7d) from an initial diameter *D*_*i*_ to the desired final diameter *D*_*f*_. These deformations are similar to pressure‐causing deformation to block an obstruction or the observation of slime deformed by gravity when placed in a beaker. We used two hyperelastic models (neo‐Hookean and Mooney–Rivlin), and a linear Hookean model for comparison to capture the large deformation of the network.

The required elastic modulus of a solid that is deforming under a particular forcing (e.g., its weight) with given geometric conditions and material properties was calculated and visualized with each model of varying complexity and fidelity (see Supporting Information for details). As an example use case, consider a material with typical parameters for an incompressible elastic material, ρ = 1000 kg/m^3^, ν = 0.5, and Mooney–Rivlin parameter ratio *C*_10_/*C*_01_ = 3 (plot with *C*_10_/*C*_01_ = 10 is shown in Figure S10, Supporting Information). Figure 7c shows the required shear modulus *G* for the three models considering an initial diameter of 5 cm and height of 10 cm of the cylindrical slump. For larger deformation (smaller *D*_*i*_/*D*_*f*_), a progressively softer material is required. While this general trend is expected, the dramatic decrease in the required modulus around *D*_*i*_/*D*_*f*_ ≈ 0.65 is a noteworthy insight into from hyperelastic models. The drop‐off in required softness arises from the inherent strain‐stiffening behavior of hyperelastic materials: at large deformations, incremental increases in strain require disproportionately larger increases in stress. Specifically, of these models, the Mooney–Rivlin model is the most realistic, applicable to large deformation, and shows the most dramatic drop‐off. While in small deformations, all models are identical and only begin to deviate as deformation becomes more significant.

The required elastic modulus depends on the initial geometric conditions. For different starting heights, the required maximum elastic modulus is different and decreases as the height decreases (Figure S11, Supporting Information). With gravity forcing, a lower initial height results in lower stresses for the same diameter ratio, which decreases the required modulus of the material even further. Figure 7d shows the modulus requirements for three different models subjected to 10 N compressive force, a nominal force that might occur in clogging, e.g., due to stagnation pressure $P=\frac{1}{2}\rho v^{2}$ of water flowing at a velocity of 3 m/s into a slime mass of diameter 5 cm. A lower compressive force would require a softer material for the same deformation.

The dramatic drop in required modulus (around *D*_*i*_/*D*_*f*_ ≈ 0.6) occurs for both hyperelastic models, regardless of whether deformation is driven by gravity or compression. Beyond this point, further reductions in modulus yield diminishing returns: a modulus of 100 Pa is nearly sufficient, while 10 Pa offers only marginal additional deformation. Thus, a conservative lower‐bound target of 10 Pa may be adequate for achieving extreme deformation in hyperelastic materials. However, this threshold may shift with more complex models, particularly when capturing the unusually large deformations seen in hagfish slime. Similar “lock‐out” behavior is observed in fibrous biological tissues such as arteries,^[^ ^42^, ^43^ ^]^ where being soft is beneficial only up to a point, beyond which further deformation becomes limiting or detrimental. If rapid deformation is required at short timescales, viscous effects may become a relevant secondary criterion. One way to assess this is by comparing the targeted deformation timescale to the viscoelastic retardation time of the material. For example, in the simplest Kelvin–Voigt model, the viscoelastic retardation time, relevant for an applied‐stress scenario, is given by the ratio of viscosity to modulus. As an example, with a modulus of 10 Pa and a water‐like viscosity of 10^−3^ Pa·s, the retardation time is τ = η/*E* = ∼10^−4^ s, which is 1000 times shorter than the ∼0.4 s timescale of the attack. As the modulus decreases, the viscoelastic timescale becomes even shorter. Thus, in our system, we neglect this effect; however, viscous contributions become important once the retardation time approaches or exceeds the required deformation time of the material. In such cases, rate‐dependent and viscous effects will act together to resist deformation. To further refine design criteria, we next examine the required properties of individual threads within such networks and how those influence bulk performance.

### 5.2 Results: Average Individual Thread Properties Requirements

We build upon an existing structure‐property model^[^ ^18^ ^]^ and invert it to understand the “design” perspective: given a continuum modulus target (e.g., *G* = 10 Pa), this may be achieved with a network of fibers of various fiber density *n*, fiber diameter *d*_*f*_, fiber modulus *E*_*f*_, and interestingly, remnant “slack” in the fiber network *h*/*l*.^[^ ^10^, ^18^ ^]^

**Figure 8:**
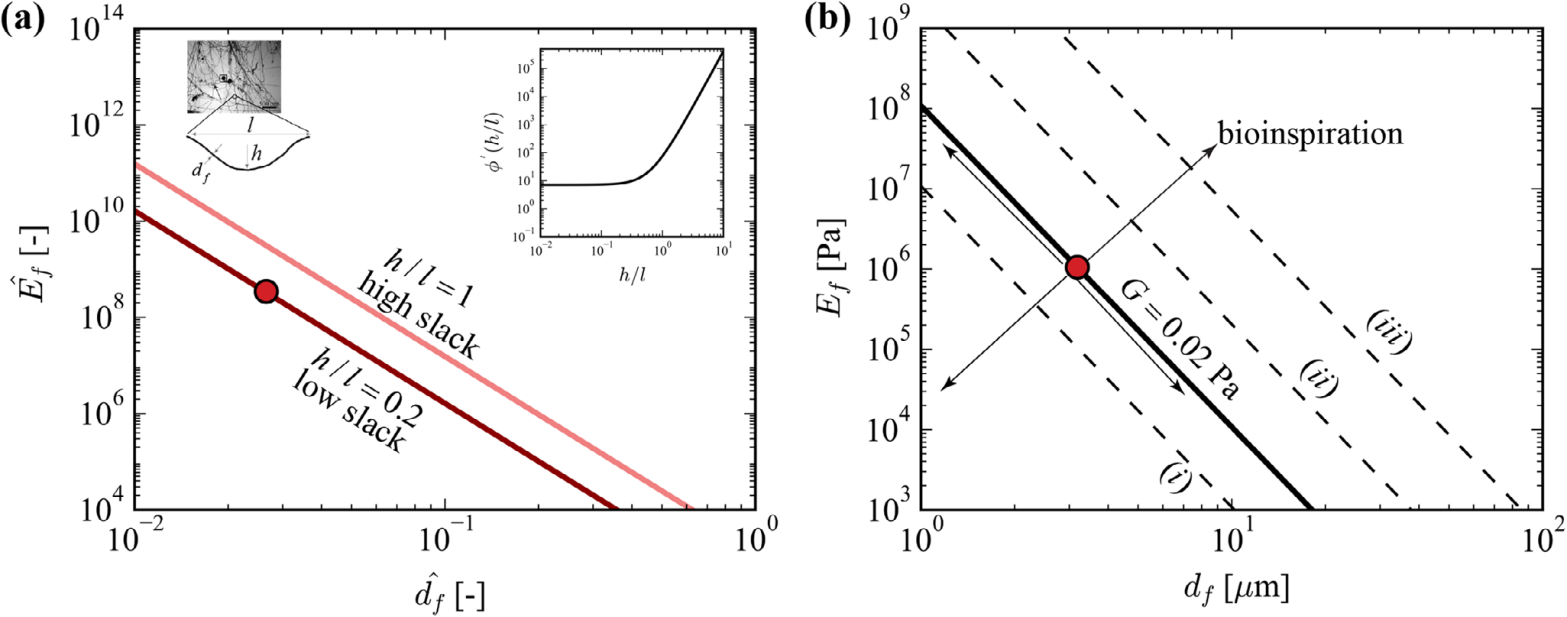
Individual thread properties requirements. a) Nondimensional fiber modulus ${\hat{E}}_{f}$ as a function of nondimensional fiber diameter ${\hat{d}}_{f}$ for different ratios of *h*/*l*. The inset shows the remnant curvature function ϕ′(*h*/*l*), Equation 15. b) Design guides (trade‐off pathlines through the design parameter space) for different network properties. Required maximum fiber modulus for biomimetic hagfish network properties (red symbol) with *G* = 0.02 Pa, *n* = 1.6 · 10^7^ m/m^3^, *h* = 23 µm, and *h*/*l* = 0.2. Bioinspired tradeoff (solid lines) with varying *d*_*f*_. Bioinspiration tradeoff pathlines for other conditions, changing one parameter to deviate from natural conditions (dashed lines): (i) higher network density, *n* = 1.6 · 10^8^ m/m^3^, (ii) more slack, *h* = 100 µm, and (iii) stiffer continuum, *G* = 10 Pa.

The structure–property relationship models individual fibers as athermal, cylindrical elastic rods of diameter *d*_*f*_, neglecting thermal contributions during deformation. We briefly summarize the model^[^ ^18^ ^]^ and then discuss inversion to inform design requirements. Under applied strain, fibers bend and straighten, but due to geometric constraints, fibers are nearly inextensible and undergo minimal axial stretch unless fully uncoiled. The shear modulus of an isotropic 3D network of such fibers is derived, assuming linear elasticity, by equating the bending energy per unit volume to the strain energy per unit volume, yielding

(12) $$G=\frac{\pi }{64}nd_{f}^{4}E_{f}\langle {\left({\left.\frac{d\kappa }{d\gamma }\right|}_{\gamma =0}\right)}^{2}\rangle$$

where *n* is the total length of all fibers per unit volume and κ(γ, *s*) is the curvature of the fiber (γ is the applied shear strain and *s* is the curvilinear distance along the fiber). κ(γ, *s*) was approximated using a Taylor series expansion about the natural curvature, resulting in the above expression. The term in angular brackets was evaluated by purely geometric arguments utilizing the inextensibility of the fibers in the limit of small strain and the resulting network shear modulus becomes

(13) $$G=\frac{\pi }{64}\frac{nd_{f}^{4}E_{f}}{h^{2}}\varphi \left(\frac{h}{l}\right)$$

where *l* is defined as the end‐to‐end length of a curl in a fiber, i.e., the half‐wavelength of naturally curved fiber, and *h* is the amplitude of the curl perpendicular to the end‐to‐end vector (inset of **Figure** **8**a). The network modulus is directly proportional to the bending stiffness of the fiber ($G∼E_{f}d_{f}^{4}$).

To understand the design perspective, Equation 13 is inverted, and the required individual fiber properties are calculated as a function of the required network properties from

(14) $$E_{f}=\frac{64G\;h^{2}}{\pi n\;d_{f}^{4}}\varphi^{\prime }\left(\frac{h}{l}\right)$$

where

(15) $$\varphi^{\prime }\left(\frac{h}{l}\right)=\frac{1}{\varphi \left(\frac{h}{l}\right)}=\frac{64{\left(1+4{\left(\frac{h}{l}\right)}^{2}\right)}^{4}}{{\left(3+20{\left(\frac{h}{l}\right)}^{2}\right)}^{2}}.$$

To remain soft, we impose *G* = *G*_max _, resulting in a maximum *E*_*f*_ allowed. The required fiber modulus *E*_*f*_ is inversely related to fiber diameter *d*_*f*_, and is influenced by the dimensionless ratio *h*/*l*, which captures remnant curvature in extended fibers (microscope image in Figure 1b). The function φ′ has weak dependence for *h*/*l* < 1 and greater sensitivity at higher values where fibers retain more slack in the network. In this regime, increased slack enhances network deformability, allowing the potential use of stiffer fibers without compromising network softness.

Figure 8a plots the dimensionless fiber modulus $\hat{E_{f}}=E_{f}/G$ versus dimensionless diameter $\hat{d_{f}}=n^{\frac{1}{4}}d_{f}/h^{\frac{1}{2}}$ for varying *h*/*l*. Greater remnant curvature lowers the network modulus *G*, or conversely, permits higher *E*_*f*_ for a fixed network softness, providing a useful design lever for tuning 3D fiber networks.

We construct design maps with trade‐off pathlines in a parameter space that relates fiber modulus *E*_*f*_ to diameter *d*_*f*_ for networks with specified properties. The key insight is that network behavior is governed by the fiber bending stiffness, linking *E*_*f*_ and *d*_*f*_. For a network that mimics hagfish slime–with *G* = 0.02 Pa, *h* = 23 µm, and fiber density *n* = 1.6 × 10^7^ m/m^3^, the red dot in Figure 8b corresponds to the fiber modulus *E*_*f*_ and diameter *d*_*f*_ of hagfish threads. The solid diagonal line represents all (*E*_*f*_, *d*_*f*_) combinations that yield the same network modulus for these conditions. The dashed lines illustrate how this shifts under different design needs: (i) higher network density moves it down, (ii) increased fiber curvature (slack) shifts it up, and (iii) targeting a higher network modulus also shifts it up. These trade‐offs are contained in the high‐dimensional design equations, Equations (14), (15).

The design maps in Figure 8 cannot be experimentally validated within this work, as true network‐level tests would require a large number of skeins. However, our framework is consistent with constitutive models for random networks of straight, linearly elastic fibers,^[^ ^44^ ^]^ which similarly predict that the network modulus scales with fiber density, filament bending stiffness, and geometry. Acknowledging these limitations, we do not use this model for precise prediction, but rather use it as a guide to identify the upper bound estimates on maximum feasible stiffness and diameter of the individual skein fibers to relate to the network‐level properties. Although the theory of Chaudhary *et al.* imposes no formal limit,^[^ ^18^ ^]^ cases with *h*/*L* > 1 are likely not physically realizable for circular fiber arcs, as the connection paths may be more complex than the model assumes. Thus, predictions in this regime may not be exact, though the qualitative insight that allowable fiber elastic modulus increases with *h*/*L* remains valid, and future models may refine this description.

## 6 Discussion

To guide material selection and fabrication of synthetic skeins, we consolidate the requirements on fiber modulus *E*_*f*_, diameter *d*_*f*_, and strain‐to‐break ε_break_ imposed by all four functional requirements. These requirements are interpreted in terms of both material property selection (*E*_*f*_, ε_break_) and the practical challenges of manufacturing fibers at small diameters. Among the constraints, the uncoiling strain requirement proves most restrictive for *E*_*f*_ and *d*_*f*_, particularly when targeting extreme hidden length ratios. These constraints are used to define a viable design space, and using this framework, we demonstrate the first‐ever fabrication of engineered synthetic skeins.

Previously, no fabrication method could produce soft fibers below 10 µm in diameter that can retain shape and deform significantly to unravel under flow, due to fundamental manufacturing limitations. Our recently introduced solvent exchange printing method overcomes this barrier, enabling the direct creation of ultra‐fine, shape‐stable threads with diameters comparable to hagfish slime threads (∼1.5 µm), with continuous lengths reaching tens of centimeters, and a wide range of materials with elastic moduli from 5 MPa to 3500 MPa, unlocking a capability previously inaccessible.^[^ ^27^ ^]^

### 6.1 Ashby Plot for Thread Candidates

**Figure 9:**
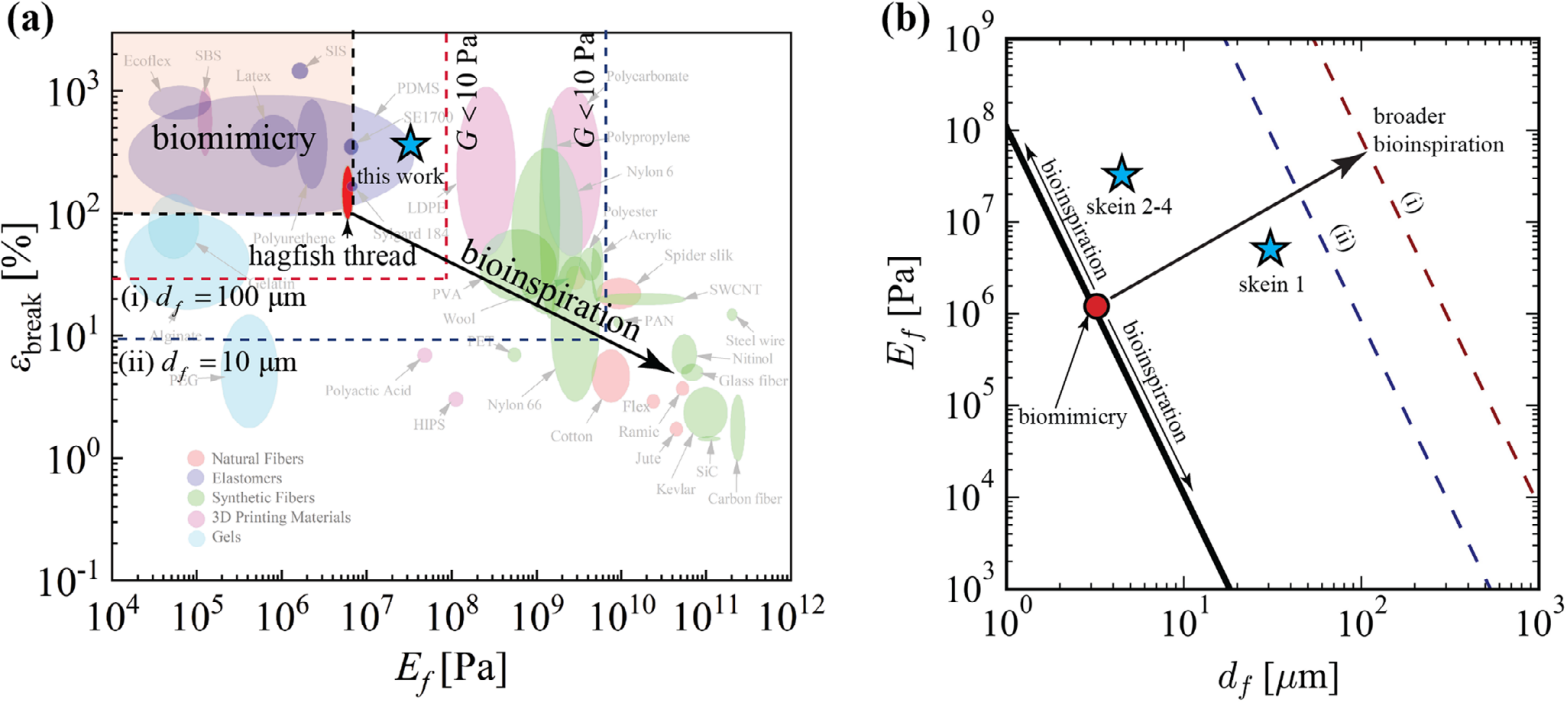
Material property requirements to achieve specific functional requirements: a) coil‐uncoil and soft network, b) soft network. Note, smaller *d*_*f*_ and softer *E*_*f*_ make every functional requirement easier to achieve. a) An Ashby‐style design diagram of elongation to break ϵ_break_ vs. Young's Modulus *E*_*f*_ of different candidate materials for synthetic skeins (full details in Supporting Information, Figure S12). Note the comparison of commercial fiber materials (e.g., Nylon 66), additive manufacturing materials (e.g., LDPE), elastomers (PDMS), and biological materials (e.g., hagfish slime thread). Design lines are shown for specific cases based on the guidelines mentioned in this paper. Horizontal dashed lines are based on coil‐uncoil functional requirement with *d*_*f*_ = 100 µm, ℜ_min _ > 200 µm and *d*_*f*_ = 10 µm, ℜ_min _ > 50 µm. Vertical lines represent the materials that can satisfy the criteria to form a soft network functional requirement with *G* < 10 Pa with (i) *d*_*f*_ = 100 µm, *h* = 500 µm and *n* = 1 · 10^5^ m/m^3^ and (ii) *d*_*f*_ = 10 µm, *h* = 50 µm and *n* = 1 · 10^5^ m/m^3^. (b) Design guides with trade‐off pathlines through the design parameter space (similar to Figure 8b), for soft network functional requirement using the same network constraints from cases (i) and (ii) in (a), but for varying *d*_*f*_ and *E*_*f*_. Star symbols represent the points corresponding to our engineered synthetic skeins (Section 6.2).

Using publicly available material property data and information from the literature,^[^ ^9^, ^45^, ^46^, ^47^, ^48^, ^49^, ^50^, ^51^, ^52^, ^53^, ^54^, ^55^, ^56^, ^57^, ^58^, ^59^, ^60^, ^61^, ^62^, ^63^, ^64^, ^65^, ^66^, ^67^, ^68^, ^69^, ^70^, ^71^ ^]^ we created a database of candidate thread materials characterized by their Young's modulus *E*_*f*_ and strain‐to‐break ϵ_break_. An Ashby‐style plot illustrating these candidate materials and their properties is shown in **Figure** **9**a (a cleaner version is provided in Figure S12, Supporting Information). This plot serves as a tool to select thread materials based on design requirements while ensuring compatibility with fabrication techniques at the required length scales.^[^ ^72^, ^73^, ^74^ ^]^ The region labeled *biomimicry* highlights materials that are simply as soft or as tough as natural hagfish threads, characterized by low modulus and high extensibility. In contrast, the *bioinspiration* region spans stiffer materials with lower extensibility that may not match natural properties exactly but can still fulfill functional requirements for deployability and soft network formation (with our engineered synthetic skeins in Section 6.2 falling within this *bioinspiration* regime). As an example, dashed lines indicate design constraints for achieving a target network modulus (*G* < 10 Pa) and fiber diameters of 100 µm and 10 µm.

Figure 9b presents a design map showing the relationship between fiber diameter *d*_*f*_ and modulus *E*_*f*_ for achieving a target mechanical behavior in synthetic skeins. The red symbol represents a design with biomimicry: matching the modulus and diameter of natural hagfish threads. The blue star symbols represent the points corresponding to our engineered synthetic skeins (Section 6.2). The labeled arrows indicate alternative design strategies: softer fibers at larger diameters, and stiffer fibers with smaller diameters (bioinspiration). The dashed lines extend this analysis to a broader bioinspired design space, using the same network constraints from cases (i) and (ii) in Figure 9a, but for varying *d*_*f*_ and *E*_*f*_.

### 6.2 First‐Ever Engineered Synthetic Skeins

**Figure 10:**
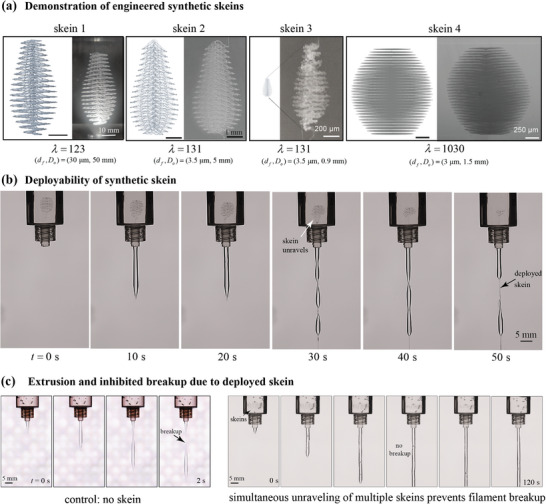
First‐ever demonstration of engineered synthetic skeins with realistic coil topology and deployability with varying geometries and packing densities, characterized by λ, *d*_*f*_, and *D*_*o*_. a) Images show skeins constructed from elastic threads with diameters ranging from 3–30 µm and packing diameters *D*_*o*_ from sub‐mm to several mm. In each case, the left image shows the CAD design, and the right image shows the printed skein in a yield stress fluid with σ_*y*_ = 48 Pa. The examples include both low and high λ, with the rightmost skein λ = 1030 approaching values seen in hagfish. b) Time‐lapse sequence showing the deployability of a synthetic skein under converging fluid flow in a yield stress fluid, σ_*y*_ = 48 Pa. Over 50 seconds, the coiled threads unravel progressively without breaking, confirming the deployability of synthetic skeins. c) Gravity‐driven extensional breakup suppressed by synthetic skeins. In the absence of skeins (left), the filament undergoes extensional breakup within 2 seconds. When multiple synthetic skeins (4) are embedded in the gel (right), their simultaneous unraveling resists extensional breakup, maintaining a continuous filament over 120 seconds.

We used bioinspired design principles to fabricate the first‐ever engineered synthetic deployable skeins using the new embedded 3D printing technique^[^ ^27^ ^]^ (strategy II, Figure 4) to achieve diameters *d*_*f*_ = 3 − 100 µm and materials with modulus *E*_*f*_ = 5 MPa (Styrene‐isoprene‐styrene (SIS), skein 1) and *E*_*f*_ = 23 MPa (Styrene–ethylene–butylene–styrene (SEBS), skein 2‐4), and ε_break_ > 300% using a series of coiled thread structures (**Figure** **10**a). Unlike strategy I, which requires a yield strength to hold the coil equilibrium state, strategy II does not rely on external yield strength to preserve the coiled structure. These synthetic skeins have packing diameters from *D*_*o*_ = 0.9–50 mm, achieving hidden length ratios from λ = 123 to λ = 1030. The first skein, generated using complex skein topology (SI: complex topology), with a relatively large packing diameter (*D*_*o*_ = 50 mm) and a fiber diameter roughly 10 times larger than natural skeins, represents a bioinspired design. In contrast, the final design (see SI: spiral skein topology) achieves a natural‐skein‐like hidden length ratio, λ = 1030, while maintaining a packing diameter only 10 times greater than natural counterparts (Figure S13, Supporting Information). All designs are still *bioinspired* and direct comparison with hagfish thread is summarized in Table S1 (Supporting Information).

We subject a bioinspired synthetic skein (spiral skein topology, *d*_*f*_ ≈ 100 µm, *E*_*f*_ = 23 MPa, λ = 108, and Figure S13, Supporting Information) to flow‐based deployment tests (Figure 10b) to assess fluid‐mediated unraveling functionality (Figure 10b; Figure S14 and Movie S2, Supporting Information). Time‐lapse imaging confirms that the coiled threads unravel progressively in a pressure‐driven extensional flow (also in simple shear flow with a lower bound estimate of EP≈50, Figure S15, Supporting Information), validating their ability to undergo controlled coil–uncoil transitions. Compared to Figure 6, which shows a single‐layer coil (*m* = 1), Figure S15 (Supporting Information) demonstrates that unraveling is still successful even with significant infill in simple shear flow, without breaking the filament. This behavior demonstrates that unraveling under fluid forces can be achieved even with larger, more manufacturable fiber diameters and is consistent with the design maps presented earlier (Figure 9), which show that bioinspired skeins can be tuned through trade‐offs among fiber stiffness, diameter, and packing geometry to satisfy functional requirements. Exploration of the soft network functional requirement is beyond the scope of the present work and offers a potential direction for future research.

While observing fiber deployment, we discovered that uncoiled skeins act to suppress the breakup of the extruded gel filament against gravity and capillary forces (Figure 10c). In control experiments without skeins, the filament undergoes rapid thinning and breaks within seconds due to a combination of extensional flow under gravity and capillary forces (Movie S3, Supporting Information).^[^ ^75^ ^]^ In contrast, embedding four skeins (λ ≈ 30, *E*_*f*_ = 23 MPa, *D*_*o*_ = 4.5 mm) and extruding the gel at 1 mm/s, the skeins unravel simultaneously and align along the filament axis, preventing breakup and maintaining filament continuity for over 120 s, at which point the skeins are fully deployed (Figure S16 and Movie S4, Supporting Information). We can rationalize the suppression of breakup with a mechanical force balance. Without fibers, the gravitational body force on the fluid is locally balanced by tensile fluid stress over the cross‐sectional area of the fluid filament, and the filament breaks up when the resulting stress exceeds the yield stress of the fluid. Assuming gravity‐driven breakup and neglecting capillary effects, the stable filament length before pinching can be estimated from $\sigma_{y}=\rho gl/\left(1.5\sqrt{3}\right)$ ^[^ ^36^, ^75^ ^]^ which for our situation predicts a critical length *l* ≈ 13 mm, which is reasonably close to the experimentally observed breakup length of approximately 20 mm (the longer length may occur due to the speed of the extrusion flow). While capillary‐driven breakup is also possible, its effect appears secondary in this system, potentially due to the dominance of yield stress over capillary pressure at the relevant length scales.

The presence of the fibers changes the force balance, i.e. the gravitational body force on the fluid can be transmitted to the fibers through shear stress at the fiber–fluid interface, enabling the uncoiled threads to bear the tensile load and arrest further thinning. This behavior is also illustrated in Figure S17 (Supporting Information), which demonstrates horizontal extrusion of a yield‐stress fluid with and without an embedded synthetic skein (λ ≈ 100, *E*_*f*_ = 23 MPa, *D*_*o*_ = 6 mm) in a gap‐spanning geometry (Movie S5, Supporting Information). Extrusion of the yield‐stress medium alone (σ_*y*_ = 48 Pa) results in filament breakup when the gravitational stress exceeds the yield stress, preventing the formation of stable gap‐spanning bridges (left column of Figure S17, Supporting Information). When a synthetic skein with a hidden length ratio λ ≈ 100 is embedded within the same medium, unraveling of the fiber during extrusion reinforces the deposited structure and enables gap‐spanning (right column of Figure S17, Supporting Information). While Figure 10c demonstrates that skeins can stabilize extensional flow by maintaining a fiber under tension, the threshold skein concentration and the role of thread wettability in pinch‐off dynamics remain open questions for future study. Much more complexity may be involved in this remarkable behavior, but these results show that skein deployment reinforces the surrounding gel under tension, hinting at potential mechanisms in the natural defense gel and offering a new paradigm for functional material design inspired by hagfish slime.^[^ ^10^, ^15^ ^]^ This research may open several promising directions for future work and potential industrial applications that may use the uncoiling of synthetic skeins to form soft solid networks. This may include oil‐drilling safeguards (plugging or slowing leaks with small amounts of pre‐deployed material), defense applications (clogging or tangling intakes with rapidly expanding fibers), biomedical uses (providing fibrous scaffolds for tissue culture at unique length scales), and manufacturing of non‐woven materials through novel unraveling‐based processes. While inspired by the hagfish system, the principles are broadly relevant to other soft biological and synthetic materials.

## 7 Conclusion

To engineer rapidly deployable soft materials inspired by hagfish slime, this work establishes design principles for synthetic skeins used to create the first‐ever deployable synthetic skeins. Thin and soft biomimetic fibers achieve all functional requirements. However, we showed that broad bioinspired design is possible, i.e., large stiff fibers can also achieve these desired functional requirements, making fabrication possible with current and recently developed techniques.^[^ ^26^, ^27^ ^]^ Design trade‐offs exist among the available materials, balancing thread performance with manufacturability constraints, ultimately guiding material selection for thread fabrication.

Key parameters to consider include fiber diameter (*d*_*f*_), packing diameter (*D*_*o*_), hidden length ratio (λ), fiber elasticity (*E*_*f*_), minimum radius of curvature in the coil state (ℜ_min _), fiber total length (*L*_*f*_) and the strain‐to‐break limit (ϵ_break_). Targeting a desired hidden length ratio (λ), such as λ = 1000, requires selecting an appropriate topology that sets the necessary *D*_*o*_/*d*_*f*_ ratio and strain (ε_max _) and higher packing density reduces *D*_*o*_/*d*_*f*_, enabling more compact skeins for a given *d*_*f*_.

Our results confirm the feasibility of using yield‐stress fluids (σ_*ys*_) to maintain manufacturable fiber diameters (*d*_*f*_) in a non‐equilibrium configuration (ℜ_min _). Notably, larger *d*_*f*_, which is advantageous from a manufacturing perspective, remains feasible when combined with higher ℜ, provided that the yield strength is appropriately selected.

Fluid‐mediated unraveling can occur for a fiber with high bending rigidity ($∼E_{f}d_{f}^{4}$), provided flow strength is sufficient (according to the EP number), with unraveling accelerated by lower fiber stiffness and higher fluid viscosity. To form a soft fibrous network, the target network continuum shear modulus (*G*) guides the selection of required $E_{f}d_{f}^{4}$, network density (*n*), and remnant slack (*h*/*l*). Together, these criteria provide a comprehensive framework for designing synthetic skeins tailored to specific functional requirements.

Guided by these principles, we identified materials and a manufacturing method to fabricate synthetic skeins using SIS and SEBS polymer threads with controlled packing geometries and varying hidden length ratios. Notably, even with a relatively large packing diameter and fiber diameters nearly 10× larger than those found in nature, our designs demonstrated that synthetic skeins can achieve the functional requirements of the coil–uncoil transition, holding elastic energy in a non‐equilibrium condition. In particular, a final design with λ = 1030 approached natural skeins while using more manufacturable scales. Flow‐based deployment tests on a representative synthetic skein further confirmed the feasibility of fluid‐mediated unraveling, without breaking the fiber, demonstrating that unraveling can be initiated even with high *d*_*f*_ values. These results are consistent with the design maps and support the broader premise that bioinspired skeins can be tuned via trade‐offs in fiber stiffness, geometry, and packing to meet key functional criteria. Exploration of the soft network functional requirement remains an open direction for future work. By replicating the skein, the key structural unit of hagfish slime, we provide a foundation for future efforts to assemble synthetic skeins into full slime‐like fiber‐mucus networks.

## 8 Experimental Section

### Preparation and Characterization of Aqueous Carbopol Microgels

Carbopol (cross‐linked poly(acrylic acid), Lubrizol USA) suspensions were prepared as yield‐stress supporting media by dispersing Carbopol 980 powder in water, neutralizing to pH 7 with NaOH to induce particle swelling, and mixing overnight to ensure homogeneity (method described in detail in Hossain et al.^[^ ^28^ ^]^). Rheological measurements were conducted with a rotational rheometer (MCR 702, Anton Paar) using a parallel‐plate geometry of 25 mm diameter for Carbopol microgel (0.5 wt% and 1 wt%). Sandpaper was affixed to both plates to mitigate slip. Dynamic yield stresses (σ_*y*_) were obtained from shear‐rate sweeps ($\dot{\gamma }=10^{-3}-100\;s^{-1}$) and fitting to a Herschel–Bulkley equation of the form^[^ ^33^ ^]^ $\sigma =\sigma_{y}\left(1+{\left(\dot{\gamma }/{\dot{\gamma }}_{crit}\right)}^{n}\right)$(Figure S3, Supporting Information). The elastic modulus of commercially available polyamide/polyurethane threads was measured with a rectangular torsion fixture on an ARES‐G2 rheometer in axial mode at a Hencky strain rate of 60%/min (Figure S3, Supporting Information).

### Experimental Validation of Holding Coiled Fibers Using a Yield Stress Fluid

Experimental validation of holding coiled fibers using a yield stress fluid was carried out using commercial fibers embedded in Carbopol microgel as a model yield‐stress medium. To determine the minimum radius of curvature (*ℜ*_min _), fibers were fixed at one end and displaced at the other to successive positions before release (Figure S4, Supporting Information). The smallest stable curvature was defined as the condition that remained held in place by the gel (1.0 wt% Carbopol, σ_*y*_ = 113 Pa). Additional tests in 0.5 wt% Carbopol (σ_*y*_ = 69.6 Pa) involved bending fibers into U‐shapes, merging their ends, and then cutting to allow free relaxation.

### Experimental Validation of the Unraveling Criteria of Elastic Fibers in a Viscoplastic Medium

Coiled springs used to validate the unraveling criteria of elastic fibers in a viscoplastic medium were 3D‐printed using solvent exchange 3D printing, introduced by Eom et al.^[^ ^27^ ^]^ For the ink, 25 wt% Styrene Ethylene Butylene Styrene (SEBS) solutions were prepared by the method described in Eom et al.^[^ ^27^ ^]^ Briefly, SEBS were placed with toluene in a 300 mL two‐neck reactor and swelled for 1 h. Afterward, an overhead stirrer was used to stir at 300 rpm while refluxing for 5 h at 120 °C. During this time, a condenser was installed in the reactor to prevent evaporative loss of the toluene. Next, it was cooled at room temperature for 6 h and then stored in a container for use. An ethanol–water suspension of Carbopol microgel particles was prepared as the supporting gel, with 70 wt% ethanol and 30 wt% water by the method described in Eom et al. Carbopol particles dispersed in the ethanol–water mixture initially formed a viscous suspension at pH ∼4. Neutralization with triethanolamine induced particle swelling, producing a dense, jammed suspension with yield stress due to electrostatic repulsion and steric interactions among the negatively charged particles. Maximum swelling occurred near pH 7, yielding a soft, glassy suspension with high viscosity and yield stress. The formulation was homogenized by overnight stirring at 200 rpm with an overhead mixer, followed by degassing in a planetary centrifugal mixer (THINKY) prior to rheological characterization and 3D printing. The rheological properties of polymer inks and gels were analyzed by using rotational rheometers (DHR‐3 and ARES‐G2; TA Instruments). All printing was carried out using a customized commercial 3D printer (TAZ Pro, LulzBot) equipped with a dispenser (Ultimus V; Nordson).

### Engineered Synthetic Skeins Using Embedded 3D Printing by Solvent Exchange (3DPX)

Styrene–ethylene–butylene–styrene (SEBS) and Styrene‐isoprene‐styrene (SIS) were selected as printing materials because they are among the most well‐studied systems for the solvent‐exchange 3D printing (3DPX) method,^[^ ^27^ ^]^ offering reliable control over solidification and thread dimensions. In principle, other materials can also be employed by building on the solvent‐exchange framework described by Eom et al.^[^ ^27^ ^]^ For synthetic skeins, the ink medium contained 52 wt% SIS and 25 wt% SEBS, extruded into an ethanol‐based yield‐stress gel. During deposition, solvent (toluene) diffuses out of the ink into the gel, while the non‐solvent (ethanol) penetrates the ink, inducing rapid phase separation and solidification at the filament surface. This solvent‐exchange process proceeds at rates up to 2.33µm/s, enabling higher extrusion speeds.

## Conflict of Interest

The authors declare no conflict of interest.

## Supporting information

Supporting Information

Supplemental Movie 1

Supplemental Movie 2

Supplemental Movie 3

Supplemental Movie 4

Supplemental Movie 5

## Data Availability

The data that support the findings of this study are available from the corresponding author upon reasonable request.
