# Supplementary material for: Design Principles for Deployable Fibers Inspired by Hagfish Defense
Source: Adv Sci (Weinh). 2025 Oct 7;12(48):e12414. doi: 10.1002/advs.202512414 (PMC12752651; doi:10.1002/advs.202512414)
Supplement: Supplementary file 1 — Supporting Information [file ADVS-12-e12414-s005.pdf]

Supplementary information for design principles for deployable fibers inspired by hagfish defense

Mohammad Tanver Hossain[1,2], Wonsik Eom[1,3], Pallab Layak[1], Jeongmin Kim[1], Carolyn Darling[1], Andrew Lowe[4], Douglas Fudge[4], Sameh H. Tawfick[1,2], and Randy H. Ewoldt[1,2,*]

[1]Department of Mechanical Science and Engineering, Grainger College of Engineering, University of Illinois Urbana-Champaign, Urbana, IL 61801, USA
[2]Beckman Institute for Advanced Science and Technology, University of Illinois Urbana-Champaign, Urbana, IL 61801, USA
[3]Department of Fiber Convergence Material Engineering, Dankook University, Republic of Korea
[4]Schmid College of Science and Technology, Chapman University, Orange, CA 92866, USA
[*]Correspondence: ewoldt@illinois.edu

1 Complex topology

To illustrate how the nested cylindrical coil topology functions, we first analyze the simplest case of a multi-layered cylindrical skein. The hidden length ratio is estimated from the analytical expression

$$\lambda_{\max} = \frac{\pi}{4}\left(\frac{D_o}{d_f}\right)^2 \left\{1 - \left(\frac{\Re_{\min}}{D_o/2}\right)^2\right\}, \tag{1}$$

where D_o is the skein diameter, d_f is the fiber diameter, and $\Re_{\min}$ is the minimum radius of curvature of the trajectory. For the example in Figure S1(a), we use $D_o = 6$ mm, $d_f =$

0.4 mm, and $\Re_{\min} = 1.8$ mm, which gives $\lambda_{\max} \approx 113$.

The X–Z plane projection highlights how individual cylindrical loops are connected by straight bridging segments, resulting in a continuous nested architecture. Beyond this idealized cylindrical case, Figure S1(b–c) shows more complicated skein-like trajectories generated by varying pitch and radius functions, yielding hidden length ratios of $\lambda = 101$ and $\lambda = 787$, respectively. The design is based on the following parametric equations to a topology inspired by coiled hagfish threads

$$\theta = t, \quad \rho = a \cdot \cos(n \cdot \theta), \quad z = h \cdot \exp\left(-\frac{\rho^2}{2c^2}\right) + p \cdot \theta, \tag{2}$$

where:

- t: Angular parameter over the helix period.
- ρ: Radial position defined by the radius function a and the tightness factor $n = (k-1)/k$.
- z: Vertical position adjusted for the pitch p and Gaussian modulation based on ρ and $c = a/c_1$.
- a: Radius function obtained by fitting a cubic spline to the predefined control points (t_1, r_1).
- $p = \frac{\mathrm{dp}}{\pi}$: The pitch, defined by the parameter dp, which controls the spacing between coils.
- c_1: Scaling constant for the Gaussian modulation.
- H, h: Overall height and local height adjustment of the topology, respectively.

The Cartesian coordinates are derived from the polar coordinates: $x = \rho \cdot \cos(\theta), \quad y = \rho \cdot \sin(\theta)$. Two sets of parameters are compared to demonstrate the impact on the topology: (1) $dp = 0.062$, $step = 0.001$ and (2) $dp = 0.008$, $step = 0.01$. Hidden length ratio (λ) of the topology is calculated from $\lambda_{\max} = s/H$, where s is the total arc length of the helical path and $D_o = H = 6$ mm is the outer diameter of the topology. The minimum radius of curvature is approximately 0.532 mm and 0.491 mm, respectively, for $\lambda_{\max}$=101 and 787. The minimum spacing between adjacent segments along the curve is approximately 103 μm and 11 μm. To prevent self-intersection of the fiber during coiling, the maximum allowable fiber diameter should therefore be less than this spacing.

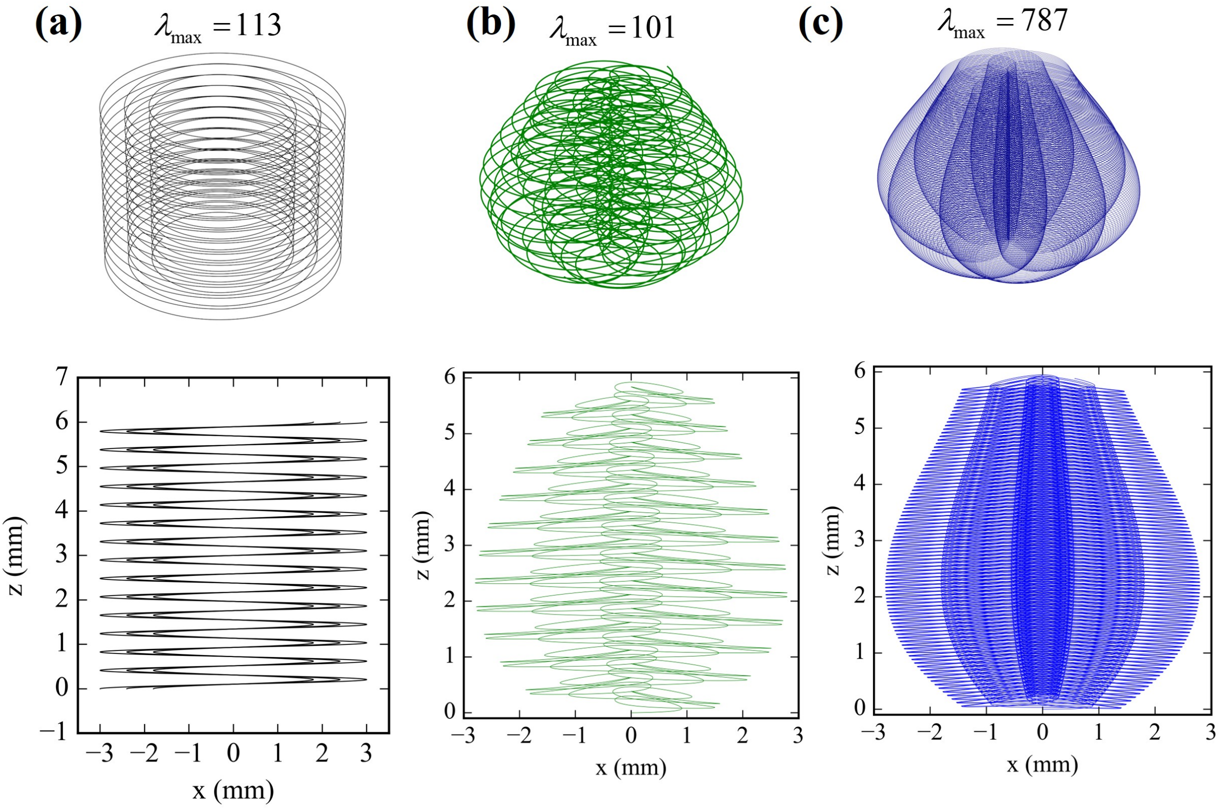

Figure S1: Synthetic skein architectures generated using the proposed framework. (a) Illustration of a nested cylindrical coil architecture ($\lambda_{\text{max}} = 113$). Each cylindrical layer is formed by a closed loop of thread. The layers are connected by straight segments that bridge from the end of one loop to the beginning of the next, producing a continuous nested structure. (b–c) Complex skein-like topologies generated using parametric equations and numerical interpolation, inspired by natural skeins. Varying pitch and radius yield different hidden length ratios ($\lambda_{\text{max}} = 101$ in b, $\lambda_{\text{max}} = 787$ in c), with $\lambda = L_f/H$, where L_f is the total arc length of the helical path and $H = D_o = 6$ mm.

2 Required yield strength to hold non-equilibrium state

Figure 5a and 5b illustrate a bent fiber of diameter d_f embedded in a yield stress fluid. For nonlocal effects, we assume a uniformly distributed resultant load (Figure 5b) acting over a length δ. We assume the resultant force is acting on the fiber as $F = \sigma_{ys} A = \sigma_{ys}(\delta.d_f)$. The resisting moment generated by the surrounding gel is

$$M_{\text{resist}} = F \cdot \frac{\delta}{2} = \sigma_{ys} \cdot (\delta \cdot d_f) \cdot \frac{\delta}{2} \tag{3}$$

Equating the moments and solving for σ_{ys} gives

$$\sigma_{ys} = \frac{\pi}{32} \cdot \frac{E_f d_f^3}{\delta^2 \Re} \tag{4}$$

and taking $\delta = \Re$, $d_f << L$ and $\delta = L/2$ results in Eq. 7 and 8 in the main text. For $\delta = \Re$

$$\sigma_{ys} = \frac{\pi}{32} \cdot \frac{E_f d_f^3}{\Re^3} \tag{5}$$

For $\delta = L/2$ we have

$$\sigma_{ys} = \frac{\pi}{8} \cdot \frac{E_f d_f^3}{\Re L^2} \tag{6}$$

3 Yield strength requirements during plastic deformation of the fibers

If the fibers undergo plastic deformation during the coil-uncoil transition, yield stress fluids may still be necessary to counteract the gravitational forces. Unraveling can induce plastic deformation, and the yield strength will be lower compared to Eqs. 7-8. A small yield strength may still be required to hold the fiber in a non-equilibrium condition and resist gravitational sagging. For example, with manufacturing techniques like embedded 3D printing, the fiber is extruded into a support medium that is a yield stress fluid and solidifies after printing. The required yield strength of the supporting bath medium is [2]

$$\sigma_{ys} > \rho g d_f + \frac{2\Gamma}{d_f}, \tag{7}$$

to avoid capillary-induced breakup of the extruded filament (droplet formation due to Rayleigh-Plateau instability) and gravitational sagging of the filament. For $d_f \ll 10\mu$m, the required yield stress to resist gravitational sagging is quite small compared to the yield stress necessary to resist capillary breakup.

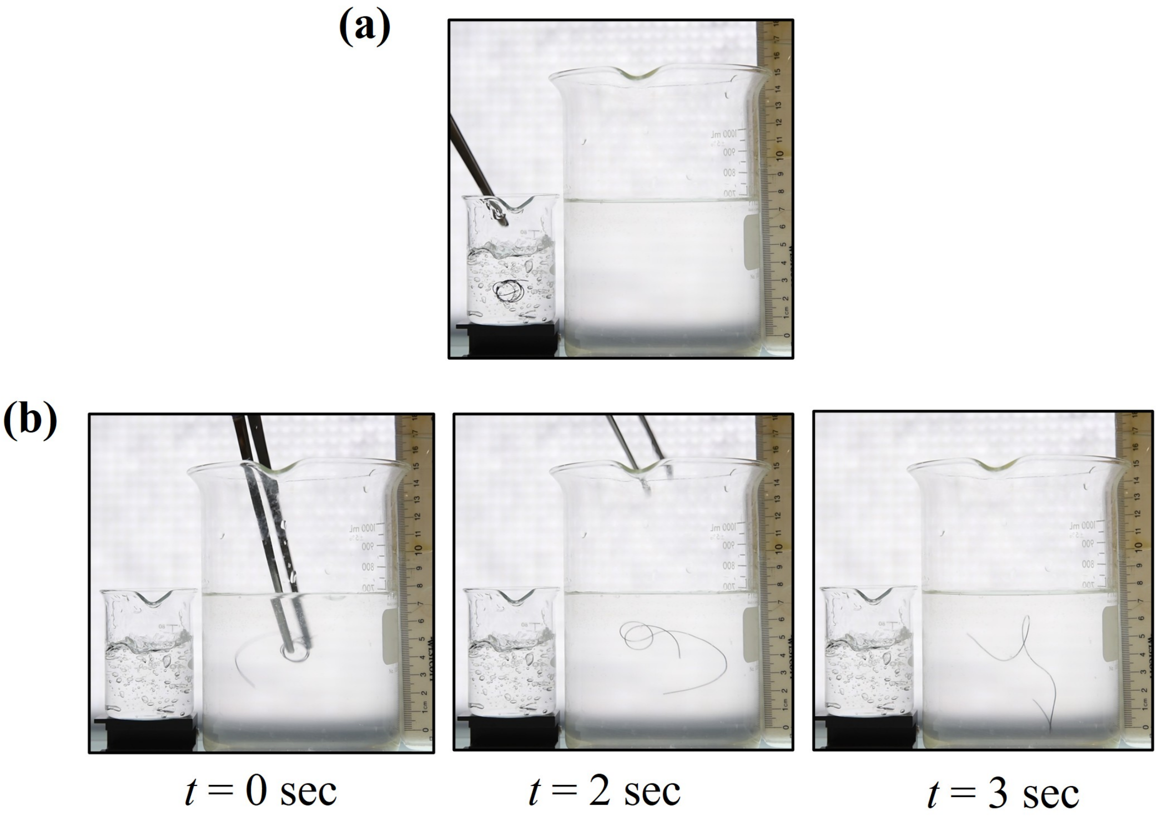

Figure S2: Demonstration of elastic recoil of a polyurethane fiber after dissolving its encapsulation in a yield stress fluid. (a) A coiled polyurethane fiber is confined within a Carbopol microgel (σ_y = 113 Pa), which maintains it in a non-equilibrium coiled state. (b) Upon transfer into a 1 wt% NaCl solution, the Carbopol dissolves due to particle deswelling, and the fiber uncoils back to its equilibrium state within 3 s. This provides a proof-of-principle that stored elastic energy can be retained in a non-equilibrium configuration by the surrounding gel and released upon dissolution of the confining matrix.

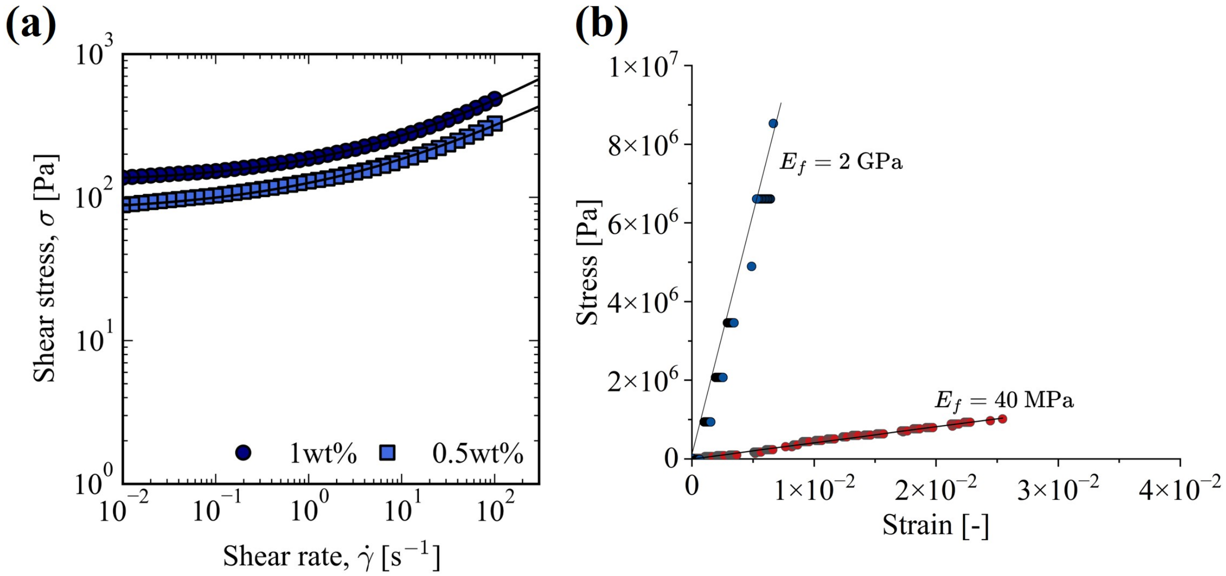

Figure S3: Characterization of yield stress fluid and elastic fibers for experiments such as those shown in Figure 5 and Figure S2. (a) Steady shear data of Carbopol 980 microgel suspensions at concentrations of 0.5 wt% and 1 wt%, showing yield stress behavior characterized by a Herschel–Bulkley model fit, obtained using an MCR-702 rheometer with 25 mm parallel plates (adapted from [3]). (b) Tensile stress–strain curves of synthetic fibers used in the skein model: polyurethane fibers with $E_f \approx 40$ MPa and polyamide fibers with $E_f \approx 2$ GPa. The Young's moduli were measured using an ARES-G2 rheometer in axial mode with a rectangular torsion fixture at a Hencky strain rate of 60%/min.

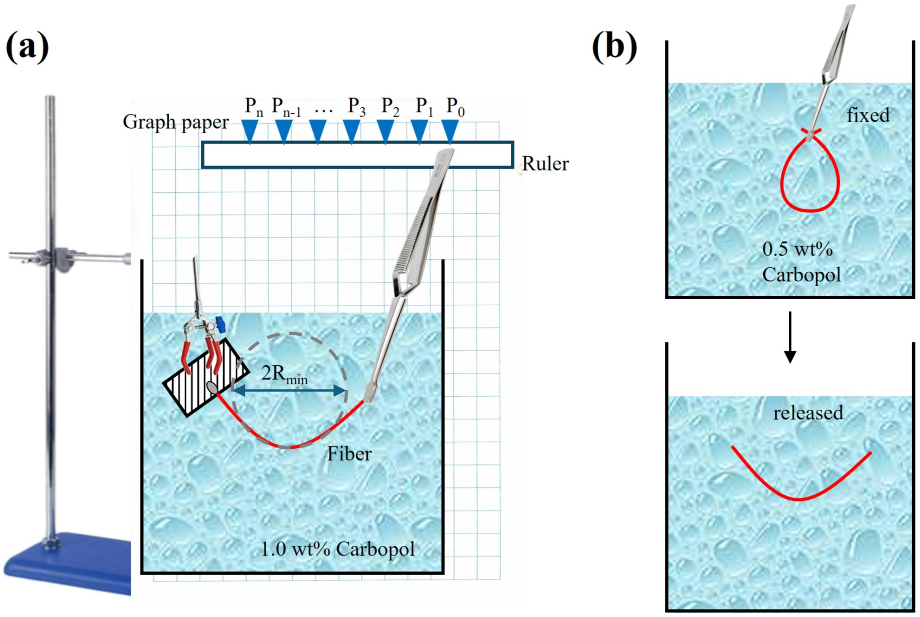

Figure S4: Experimental validation of the modeling framework using synthetic fibers embedded in Carbopol microgel as a model yield-stress medium. (a) Schematic of the setup for measuring the minimum radius of curvature of fibers in 1.0 wt% Carbopol (yield stress $\sigma_y = 113$ Pa). One end of the fiber was fixed, while the other end was displaced to successive positions marked on the background graph paper and then released. The smallest stable radius of curvature $\Re_{\min}$ was identified as the condition where it was held in place by the yield-stress medium. (b) Example test in 0.5 wt% Carbopol ($\sigma_y = 69.6$ Pa), where a fiber was intentionally bent into a U-shape by merging its two ends and then cut to allow free relaxation. The fiber relaxed into a lower-curvature configuration, determined by the balance of elastic restoring forces.

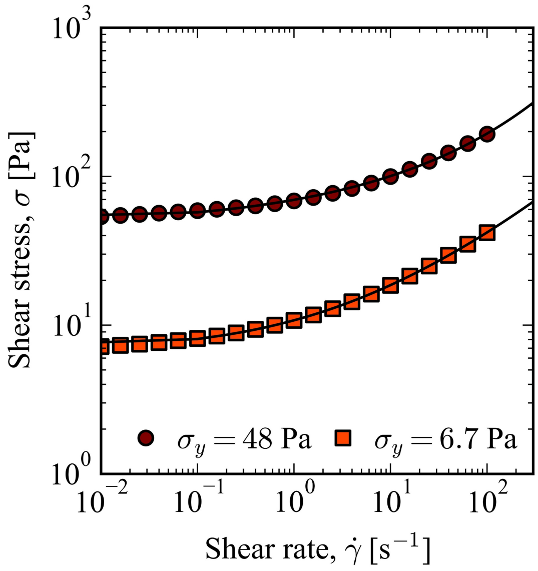

Figure S5: Steady shear flow curves of 70:30 ethanol–water gels measured on an ARES-G2 rheometer using 25 mm parallel-plate geometry. The data are fitted to a Herschel–Bulkley model, yielding dynamic yield stresses of $\sigma_y = 48$ Pa (circles) and $\sigma_y = 6.7$ Pa (squares). Adapted from [1].

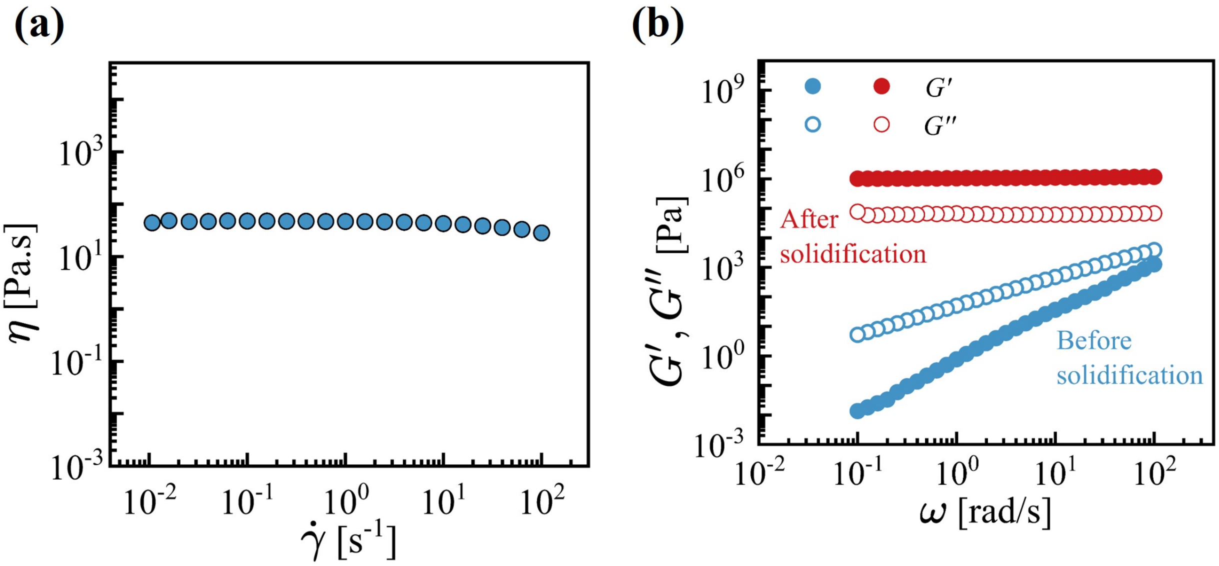

Figure S6: (a) Flow curve of a 25 wt.% SEBS solution measured in steady shear, showing nearly Newtonian behavior before solidification obtained using DHR-3 rheometer. (b) Frequency sweep of the same 25 wt.% SEBS solution before (blue symbols) and after (red symbols) solidification by solvent exchange. After solidification, the material exhibits a solid-like response with $G' \gg G''$, in contrast to the liquid-like viscoelastic spectrum observed before solidification. Adapted from [1].

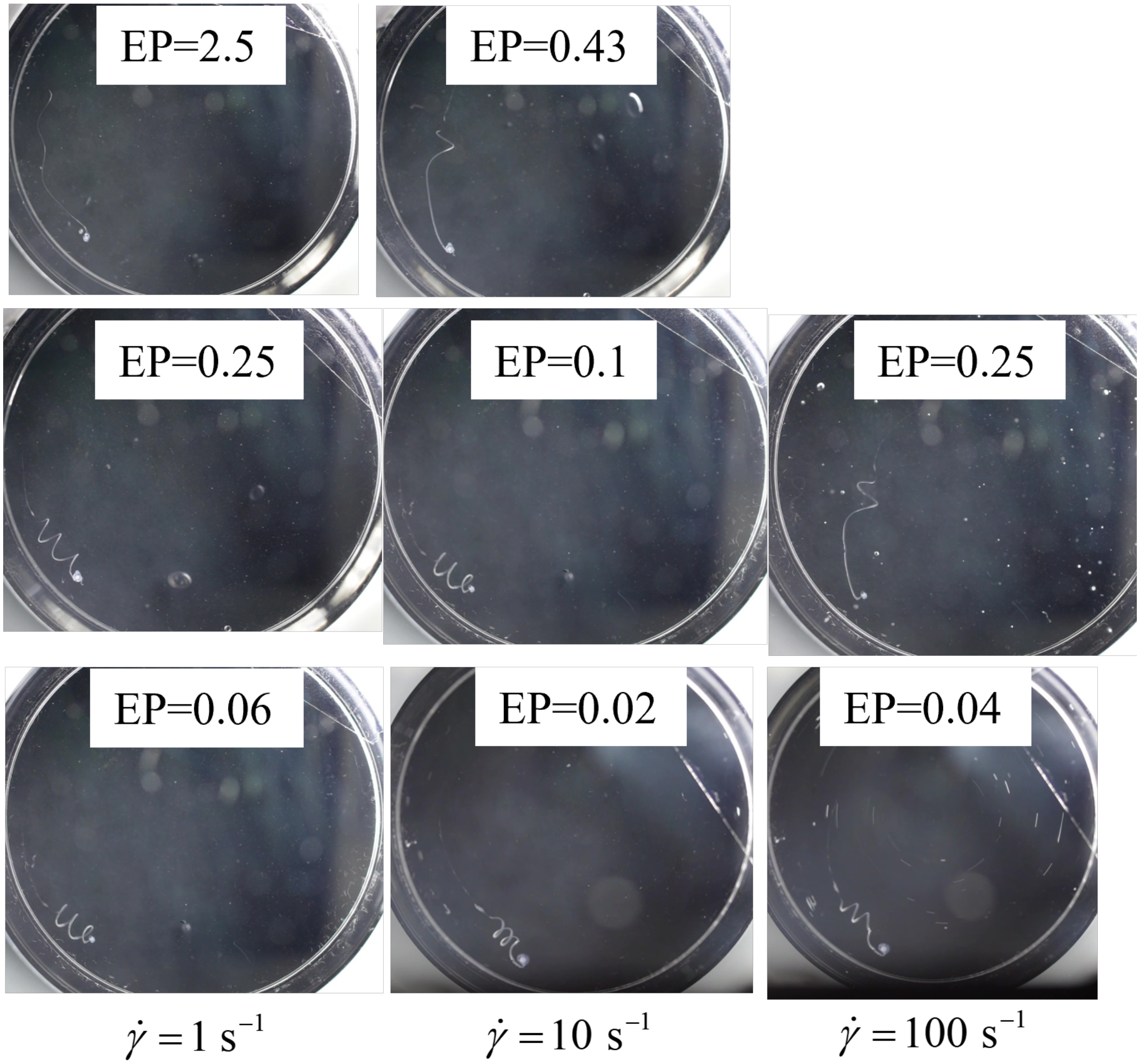

Figure S7: Unraveled state of synthetic coiled fibers after flow cessation as a function of the Elasto-Plastic number (EP). Fibers were printed in a yield stress medium and subjected to shear flow, with one end anchored to the substrate during the printing process. The final equilibrium conformations of the fibers are shown for a range of EP values, illustrating the transition from complete unraveling at high EP (top left, EP = 2.5) to partial or no unraveling at low EP (bottom row).

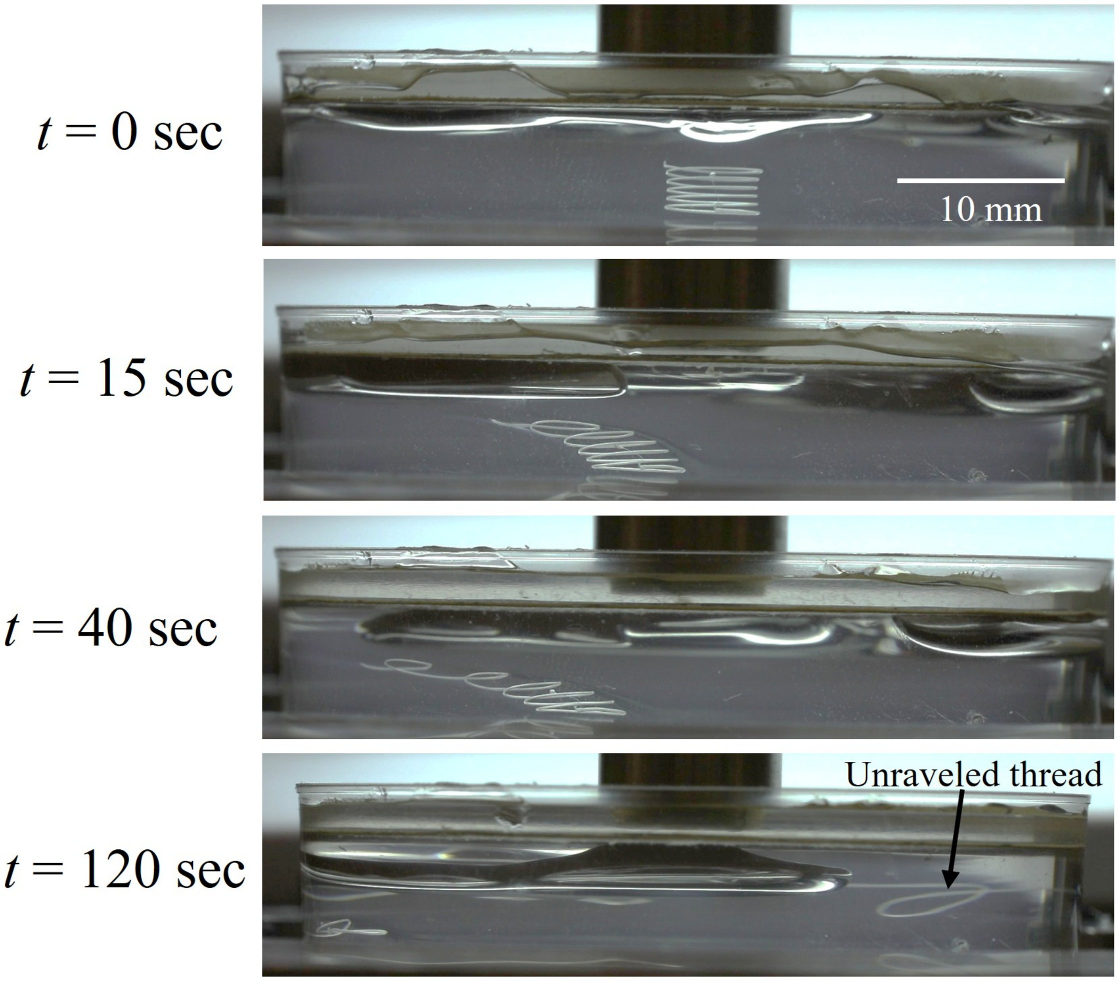

Figure S8: Fluid-mediated unraveling of synthetic coiled fibers with free ends. Time-lapse sequence shows a fiber of diameter $d_f = 200\ \mu$m and length $L = 3$ mm (EP≈ 0.6) undergoing unraveling in a yield-stress medium ($\sigma_y = 6.7$ Pa) under shear. Unlike the anchored case in Figure 6, this coiled skein was not rooted to the substrate.

4 Modeling details for unraveling timescale

If we consider the coiled rest state (strategy II), it will require forces to extend the thread, and if the elastic stored stress remains in the extended state, the threads will recoil and collapse. In this case, the unraveling time of synthetic skeins along with thread and fluid properties will also depend on the flow conditions (flow strength, flow type) as $t_{\text{unravel}} = f(d_f, L_f, \eta_s, E_f, \dot{\epsilon} \text{ or } \dot{\gamma})$.

To estimate the unraveling time for different flow conditions with affine deformation, we employed athermal bead–spring models. First, we assumed the skein was split into two parts by uniaxial extensional flow (Figure S9a), which caused unraveling. Second, we considered simple shear flow, where one part of the skein thread adheres to a surface and the other is dragged by shear forces (Figure S9b). These results are based on affine deformation assumptions and thus represent lower-bound estimates for the required flow strength. Additionally, thread adhesion (peeling force), thread elasticity, and thread inertia can increase the strain rates needed to achieve the same unraveling time. Unraveled threads may recoil and collapse when the flow stops, and we estimate a collapse time that depends on the ratio of fluid viscosity to thread elasticity, scaled by a geometric factor.

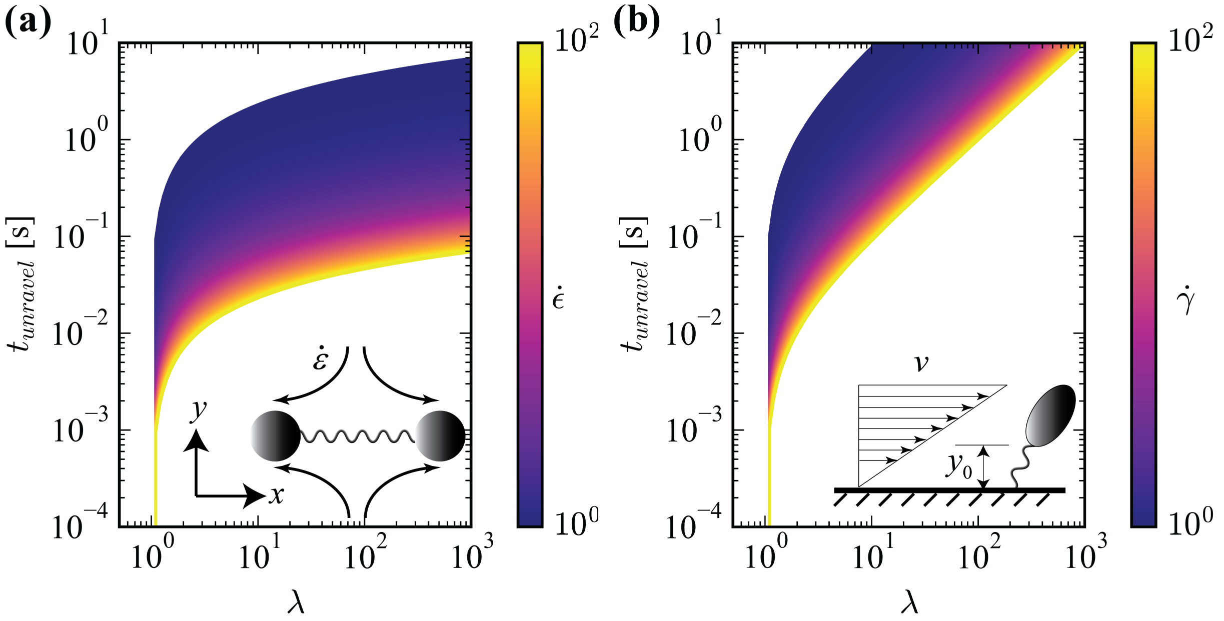

Figure S9: Unraveling time t_{unravel} as a function of hidden length ratio λ for a coiled rest state with negligible elasticity based on bead–spring model subjected to (a) uniaxial extension flow and (b) simple shear flow.

4.1 Unraveling in extensional flow

Suppose a skein is split into two parts by uniaxial extension flow, which causes unraveling, as shown in the inset of Figure S9a. For uniaxial extension flow, the extension rate is given by $\dot{\epsilon} = \frac{1}{L}\frac{dL}{dt}$, and it can be written as

$$\dot{\epsilon}_{xx} = \frac{\delta v}{\delta x} \sim \frac{\text{Velocity difference}}{\text{Distance}}. \tag{8}$$

Three key forces are acting on the skein: (i) viscous traction force on the skein from the surrounding fluid (causes unraveling), (ii) tension force within the thread (resists unraveling, e.g. due to elastic deformation), and (iii) inertia of the skein (resists the acceleration of the thread). To gain insights, the fluid-mediated unraveling of synthetic skeins is modeled using the bead spring model. The split skeins are considered as beads to model the viscous drag and inertia. The beads are connected by a spring to model the elasticity of an uncoiled skein. The governing equation that relates the three fundamental forces are given by

$$\underbrace{m\ddot{R}}_{\text{Inertia}} = \underbrace{-\xi\left(\dot{R} - [\kappa \cdot R]\right)}_{\text{Viscous Drag}} - \underbrace{2F_c}_{\text{Connector Tension}} \tag{9}$$

where R is the end-to-end length of the skein (modeled as a dumbbell), m is the mass of a single bead, ξ is the viscous drag coefficient, κ is the inverse of the local velocity gradient, and F_c is the connector tension force within the thread. To get insights, we consider a constant Stokes drag coefficient during unraveling given by $\xi = 6\pi\eta_s a$ where a is the radius of the bead and η_s is the viscosity of the surrounding fluid. The connector force F_c may be complex, however, general insight is possible by keeping F_c unspecified; we also pursue specific insight by assuming F_c arises from the elasticity of the extended thread. For uniaxial extension flow, the velocities are

$$\underline{\mathrm{v}} = \begin{bmatrix} \dot{\epsilon}x_1 \\ -\frac{1}{2}\dot{\epsilon}x_2 \\ -\frac{1}{2}\dot{\epsilon}x_3 \end{bmatrix} \tag{10}$$

and the traceless tensor κ is

$$\boldsymbol{\kappa} = \begin{bmatrix} \frac{\partial v_1}{\partial x_1} & \frac{\partial v_1}{\partial x_2} & \frac{\partial v_1}{\partial x_3} \\ \frac{\partial v_2}{\partial x_1} & \frac{\partial v_2}{\partial x_2} & \frac{\partial v_2}{\partial x_3} \\ \frac{\partial v_3}{\partial x_1} & \frac{\partial v_3}{\partial x_2} & \frac{\partial v_3}{\partial x_3} \end{bmatrix} = \begin{bmatrix} \dot{\epsilon} & 0 & 0 \\ 0 & -\frac{1}{2}\dot{\epsilon} & 0 \\ 0 & 0 & -\frac{1}{2}\dot{\epsilon} \end{bmatrix}. \tag{11}$$

In the 1D case, Eq. 9 becomes

$$m\ddot{x}_1 = -6\pi\eta_s a\left(\dot{x}_1 - \dot{\epsilon}x_1\right) - 2F_c. \tag{12}$$

As a bounded estimate on minimum flow strength, we considered the limit of negligible bead inertia, e.g. due to sufficiently high fluid viscosity as suggested by observations of natural skeins within the mucus network. In this limit, the response is affine motion of the beads with the local flow velocity. With the initial condition $L(t=0) = L_i$, we can obtain the unraveling time t_{unravel}, which is set by the kinematics of the flow and is given by

$$t_{\mathrm{unravel}} = \frac{1}{\dot{\epsilon}}\ln\left(\frac{L_f}{L_i}\right). \tag{13}$$

Figure S9a shows the unraveling time based on Eq. 13 for different values of $\dot{\epsilon}$ and unraveling can occur in less than one second given the appropriate flow strength. To have a hidden length ratio of 1:1000, i.e.L_f/L_i =1000, the time required is $t_{\mathrm{unravel}} = 6.91/\dot{\epsilon}$. The hagfish threads unravel within 400 ms. Using this very short time as a target, Eq. 13 can be rearranged to reveal a lower bound estimate of the required flow strength in terms of extensional strain rate, $\dot{\epsilon} = 17\ \mathrm{s}^{-1}$. To better understand this number, we can relate the extensional rate to velocity, $\dot{\epsilon} = \Delta v/\Delta x$. If the length scale is around 150 μm (the size of a condensed natural skein), then the velocity difference required across that length is 2.6 mm/s for the skein to unravel by a factor of 1:1000 and the final length increases to 15 cm. After obtaining a lower bound estimate on the flow strength, we can extend to consider the elasticity of the threads (a linear elastic spring is slowing down the unraveling). In this scenario, we are still neglecting the inertia of the beads, and the governing equation is given by

$$\frac{dx_1}{dt} = -\frac{H}{3\pi\eta_s a} + \dot{\epsilon}x_1 \tag{14}$$

where H is the Hook's constant of the spring. Thus, to have a hidden length ratio of 1:1000, the unraveling time required is

$$t_{\mathrm{unravel}} \sim \frac{6.91}{\dot{\epsilon} - \frac{H}{3\pi\eta_s a}} \tag{15}$$

Unraveling time will always be greater than a thread with no resistance (i.e. fully plastic thread) in Eq. 13. The elasticity of the thread (spring constant H) slows the unraveling, which may be the case if the thread has significant elasticity compared to the viscous effects. This gives a specific criteria for neglecting elasticity of the thread as $H/(3\pi\eta_s a\dot{\epsilon}) \ll 1$. Thus, even with finite elasticity of the thread, this criteria will be met for sufficiently large

surrounding viscosity and extension rate.

4.2 Unraveling in shear flow

Simple shear flow can cause the unraveling of the skein if the flow strength is sufficient. To model the shear flow unraveling, we considered a skein thread stuck on a surface, and the other part is dragged by a simple shear flow, as shown in the inset of Figure S9b. Here, y_0 is the vertical distance from the surface to the skein. Neglecting inertia and thread elasticity, the velocity of the skein is given by $\frac{dx}{dt} = \dot{\gamma} y_0$. The unraveling timescale in simple shear flow is

$$t_{\text{unravel}} = \frac{L_f - L_i}{\dot{\gamma} y_0}. \tag{16}$$

For a 1:1000 hidden length ratio in 400 ms, the shear strain rate required in this case is 2500 s^{-1}, which is two orders of magnitude higher than uniaxial extension flow. Figure S9 shows the needed unraveling time with different hidden length ratios for uniaxial extension and simple shear flow, suggesting a faster unraveling in extensional flow. Note, natural flow is a combination of both shear and extensional flow and is more complex, thus requiring a more sophisticated modeling approach to pinpoint the required flow strength.

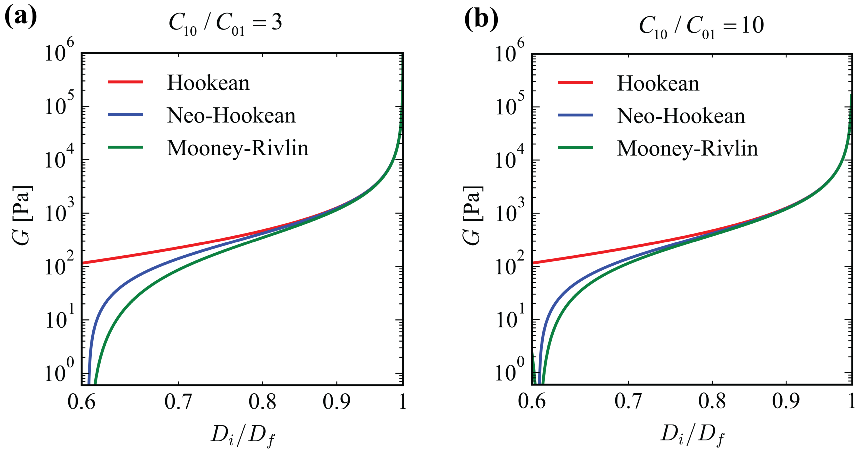

Figure S10: Required shear modulus to enable radial deformation from D_i to D_f due to gravitational forcing. (a) $C_{10}/C_{01} = 3$, and (b) $C_{10}/C_{01} = 10$ for Mooney-Rivlin. The other parameters are h_i =10 cm and D_i=5 cm, $\rho = 1000$ kg/m^3, and $\nu = 0.5$.

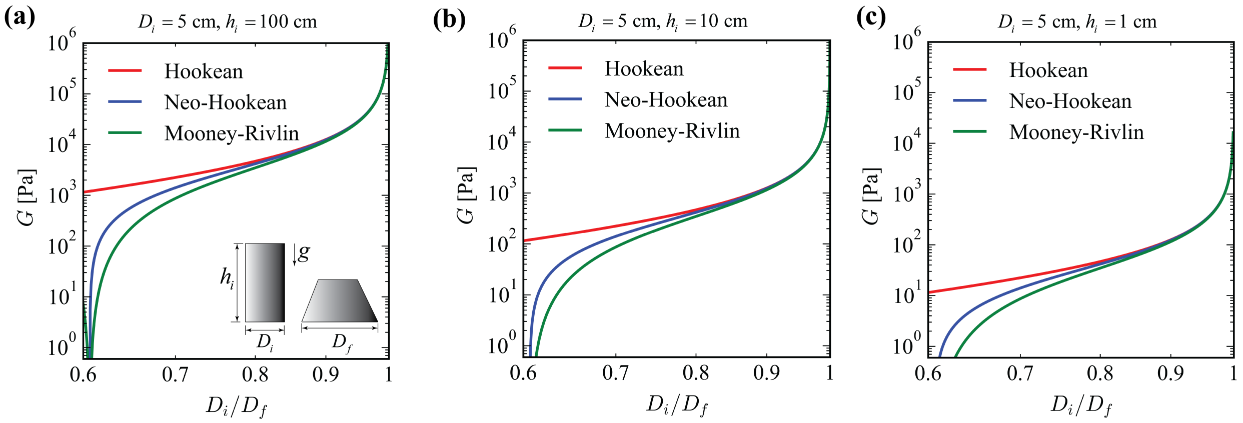

Figure S11: Required shear modulus to enable radial deformation from D_i to D_f due to gravitational forcing. (a) h_i =1 cm, (b) h_i =10 cm, and (c) h_i =100 cm. The other parameters are D_i=5 cm, $\rho = 1000$ kg/m^3, $\nu = 0.5$, and $C_{10}/C_{01} = 3$ for Mooney-Rivlin.

5 Modeling details for continuum-level material property requirements

Consider a cylindrical slime mass of height h_i and initial diameter D_i deformed under gravity or compressive force to a final height of h and diameter of at least D_f. We assume the material is incompressible (e.g. due to the large water content), isotropic (a simplifying assumption to develop insight), and subjected to uniaxial compression due to the body force of gravity.

For this inverse problem, the required modulus to achieve deformation to a final diameter of at least D_f, can be written as a function of the forcing amplitude (F_b body force due to gravity or F_c compressive force) and the geometric parameters as

$$E = f\left(F, D_i, D_f, ...\right). \tag{17}$$

For the setup shown in Figure S11a, $\sigma_{xx} = \sigma_{yy} = 0$ for any z. Neglecting accelerations, the stress distribution is determined from a force equilibrium. The normal stress on any plane at location z depends on the forcing amplitude from gravity and the cross-sectional area in the plane as

$$\sigma_{zz} = \frac{F_b}{A(z)} = \frac{V_{\text{above}}\rho g}{A(z)} \tag{18}$$

where $A(z)$ is the cross-sectional area,V_{above} is the volume above the plane, ρ is the density of the material and g= -9.81 m/s^2. Assuming linear elastic deformation (a small deformation assumption for insight), the final height h of the slump is

$$h = \frac{D_i^2 h_i}{\frac{1}{3}\left(D_f^2 + D_i D_f + D_i^2\right)}. \tag{19}$$

Diameter of any plane at a height z from the surface is

$$D(z) = \frac{\left(D_f - D_i\right)\left(h - z\right)}{h} + D_i. \tag{20}$$

Three principal strains after deformation at any plane from the surface are

$$\varepsilon_{xx}(z) = \varepsilon_{yy}(z) = \ln\left(\frac{D(z)}{D_i}\right), \tag{21}$$

$$\varepsilon_{zz}(z) = -\frac{\varepsilon_{xx}(z)}{\nu} = -\frac{1}{\nu}\ln\left(\frac{D(z)}{D_i}\right), \tag{22}$$

where $\nu = 0.5$ is taken for the Poisson's ratio for an incompressible material. For linear elastic materials, the critical elastic modulus can be obtained from the Hookean model as

$$E = \frac{\sigma_{zz}(z) - \nu\left(\sigma_{xx}(z) + \sigma_{yy}(z)\right)}{\epsilon_{zz}(z)}. \tag{23}$$

This expression gives a quantitative result for E after assuming a particular forcing (substituting in for the stress components) and a desired amount of deformation (substituting in for the strain). The shear modulus of the network then obtained using $E = 2G(1+\nu)$. However, for large deformation of the slump, the Hookean model will fail. Thus, we consider two dif-

ferent models that can capture large deformation of the slump. The first is the neo-Hookean model, a hyperelastic material model that accounts for geometric nonlinearities with large deformation and is consistent with affine rubber elasticity theory. The strain energy density function for an incompressible neo-Hookean material in a three-dimensional description is $W = C_{10}(I_1 - 3)$ where C_{10} is the material constant and I_1 is the first invariant (trace), of the right Cauchy-Green deformation tensor, i.e., $I_1 = \lambda_1^2 + \lambda_2^2 + \lambda_3^2$ where λ_i are the principal stretches. For incompressible materials and uniaxial compression, $\lambda_1\lambda_2\lambda_3 = 1$ and $\lambda_1 = \lambda$ and $\lambda_2 = \lambda_3 = 1/\sqrt{\lambda}$ and the strain can be written as $\epsilon_{zz} = \lambda - 1$. The principal stresses can be calculated from

$$\begin{aligned} \sigma_{11} = \sigma_{zz} = \lambda_1 \frac{\partial W}{\partial \lambda_1} &= 2C_{10}\left(\lambda^2 - \frac{1}{\lambda}\right), \\ \sigma_{22} = \lambda_2 \frac{\partial W}{\partial \lambda_2} &= 0, \\ \sigma_{33} = \lambda_3 \frac{\partial W}{\partial \lambda_3} &= 0. \end{aligned} \tag{24}$$

Eq. 24 can be solved as an inverse problem for the required material constant C_{10} which yields

$$C_{10} = \frac{\sigma_{zz}(z)}{2\left((\varepsilon_{zz}(z)+1)^2 - \frac{1}{\varepsilon_{zz}(z)+1}\right)}. \tag{25}$$

The required elastic modulus from the neo-Hookean model can be obtained from

$$\begin{aligned} E &= 4C_{10}(1+\nu) = 6C_{10}, \\ E &= \frac{3\sigma_{zz}(z)}{(\varepsilon_{zz}(z)+1)^2 - \frac{1}{\varepsilon_{zz}(z)+1}}. \end{aligned} \tag{26}$$

And for incompressible materials, we can write the shear modulus as

$$G = \frac{\sigma_{zz}(z)}{(\varepsilon_{zz}(z)+1)^2 - \frac{1}{\epsilon_{zz}(z)+1}}. \tag{27}$$

One must assume the forcing to determine the stress state and apply a criteria for the desired amount of deformation to determine the strain, after which quantitative targets for G and E are obtained. To account for higher-order effects beyond the neo-Hookean model, one can consider the Mooney-Rivlin model for hyperelastic materials where the strain energy density function W, is a linear combination of two invariants of the left Cauchy–Green deformation tensor written as $W = C_{10}(I_1 - 3) + C_{01}(I_2 - 3)$ where C_{10} and C_{01} both are material constants

found empirically. The principal stresses can be calculated from

$$\sigma_{11} = \sigma_{zz} = \lambda_1 \frac{\partial W}{\partial \lambda_1} = \left(2C_{10} + \frac{2C_{01}}{\lambda}\right)\left(\lambda^2 - \frac{1}{\lambda}\right),$$
$$\sigma_{22} = \lambda_2 \frac{\partial W}{\partial \lambda_2} = 0,$$
$$\sigma_{33} = \lambda_3 \frac{\partial W}{\partial \lambda_3} = 0. \tag{28}$$

Two material constants exist for this model, which makes inversion more challenging. To make progress, we solved the criteria for one of the parameters as a function of the relative value of the two parameters. Eq. 28 can then be inverted to solve for the material constants C_{01} and C_{10} which yields

$$C_{01} = \frac{\sigma_{zz}(z)}{2\left(\frac{C_{10}}{C_{01}} + \frac{1}{\varepsilon_{zz}(z)+1}\right)\left((\varepsilon_{zz}(z)+1)^2 - \frac{1}{\varepsilon_{zz}(z)+1}\right)},$$
$$C_{10} = \frac{\sigma_{zz}(z)}{2\left((\varepsilon_{zz}(z)+1)^2 - \frac{1}{\varepsilon_{zz}(z)+1}\right)} - \frac{C_{01}}{\frac{1}{\varepsilon_{zz}(z)+1}}. \tag{29}$$

which is an implicit equation for C_{01} . The value of the material constant C_{01} can be calculated assuming a range of values of the ratio C_{10}/C_{01}. Note that the material constant C_{10} becomes the same as the Neo-Hookean model for $C_{01} = 0$. The elastic modulus for the Mooney-Rivlin model is related to these model parameters allowing the above equation to be written in terms of the elastic modulus,

$$E = 4\left(C_{10} + C_{01}\right)(1+\nu) = 6\left(C_{10} + C_{01}\right),$$
$$E = 6\left[C_{10} + \frac{\sigma_{zz}(z)}{2\left(\frac{C_{10}}{C_{01}} + \frac{1}{\varepsilon_{zz}(z)+1}\right)\left((\varepsilon_{zz}(z)+1)^2 - \frac{1}{\varepsilon_{zz}(z)+1}\right)}\right]. \tag{30}$$

And for incompressible materials, we can write the shear modulus as

$$G = 2\left[C_{10} + \frac{\sigma_{zz}(z)}{2\left(\frac{C_{10}}{C_{01}} + \frac{1}{\varepsilon_{zz}(z)+1}\right)\left((\varepsilon_{zz}(z)+1)^2 - \frac{1}{\varepsilon_{zz}(z)+1}\right)}\right]. \tag{31}$$

As before, a quantitative target for softness in terms of linear modulus E depends on the strength of the assumed forcing (setting the stress) and the target amount of deformation (setting the strain).

Large compressive forces can occur during clogging events, resulting in stresses that

are much higher than in the gravitational loading considered above. Figure 7d shows the resulting deformation of a cylindrical slime mass subjected to uniaxial compressive forcing. The stress on the surface and the resultant height can be obtained from

$$\sigma_c = \frac{F_c}{A(z)}, \tag{32}$$

$$h = \frac{D_i^2}{D_f^2} h_i. \tag{33}$$

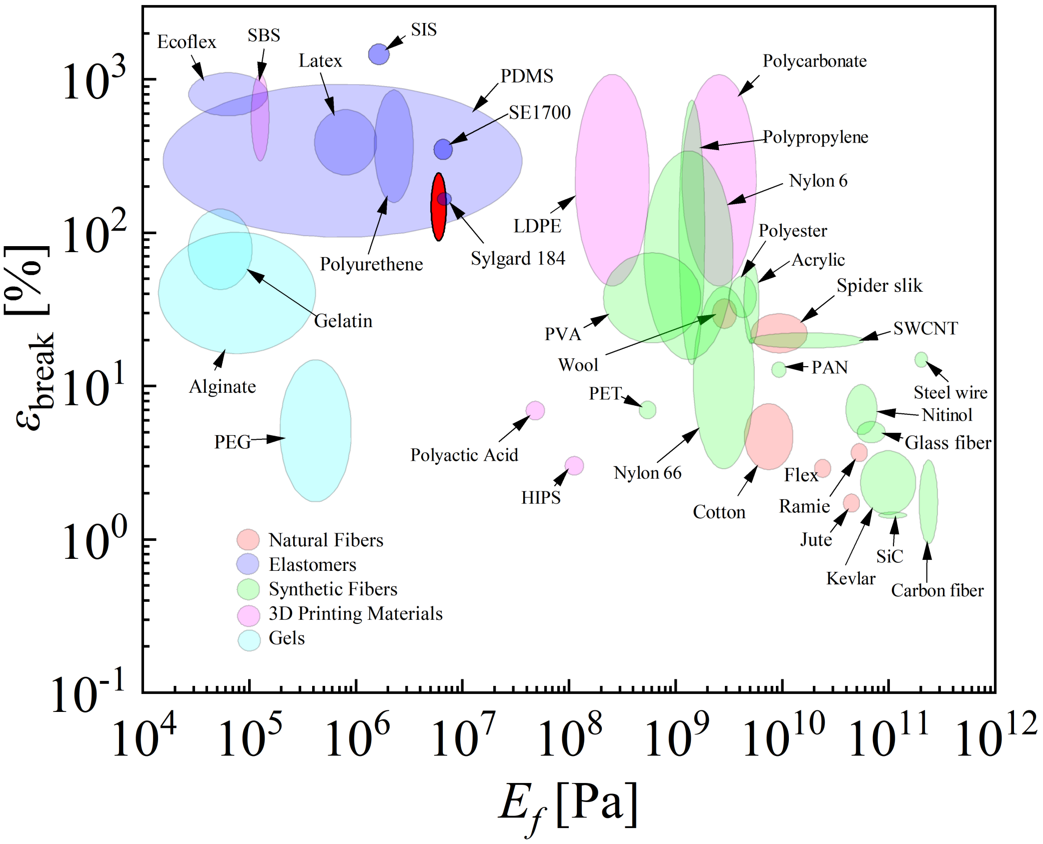

Figure S12: An Ashby-style design diagram of elongation to break ϵ_{break} vs. Young's Modulus E_f of different candidate materials for synthetic skeins. Note the comparison of commercial fiber materials (e.g., Nylon 66), additive manufacturing materials (e.g., LDPE), elastomers (PDMS), and biological materials (e.g., hagfish slime thread).

6 Spiral skein topology

The spiral skein topology was generated using alternating outward and inward spiral layers connected vertically to form a continuous fiber pathway. The design is based on the following parametric definitions:

$$r(t) = \begin{cases} \frac{w}{2\pi}\, t, & \text{odd layers (outward)} \\ a(n) - \frac{w}{2\pi}\, t, & \text{even layers (inward),} \end{cases}$$

$$x = r\cos t, \qquad y = r\sin t, \qquad z = (n-1)\, dz,$$

where:

- $t \in [0,\, 2\pi \cdot \lfloor a(n)/w \rfloor]$ is the angular coordinate over each spiral layer,
- $a(n)$ is the layer-dependent maximum radius, obtained from a cubic polynomial fit to control points (t_i, r_i),
- dz is the layer spacing, chosen such that the total height equals H,
- w is the in-plane thread spacing, which sets the number of turns in each layer.

Odd-numbered layers form spirals from the center outward to $a(n)$, while even-numbered layers return inward from $a(n)$ to the center. To enforce path continuity, layers are connected either (i) vertically at the center for inward–outward transitions, or (ii) by a short radial hop at the outer rim followed by a vertical connector for outward–inward transitions.

The total fiber length s is obtained by numerical arc-length integration, and the hidden length ratio is calculated as

$$\lambda_{\max} = \frac{s}{H}.$$

In figure S13(a), the design parameters were $H = 1.5$ mm, $dz = 50\,\mu$m, $w = 25\,\mu$m, and $N = 31$ layers. The resulting expansion ratio was $\lambda \approx 1030$. The structure provides a dense, continuous winding reminiscent of natural hagfish skeins. In figure S13(b), the design parameters were $H = 6$ mm, $dz = 0.555$mm, $w = 0.35$mm, and $N = 12$ layers. The resulting expansion ratio was $\lambda \approx 108$.

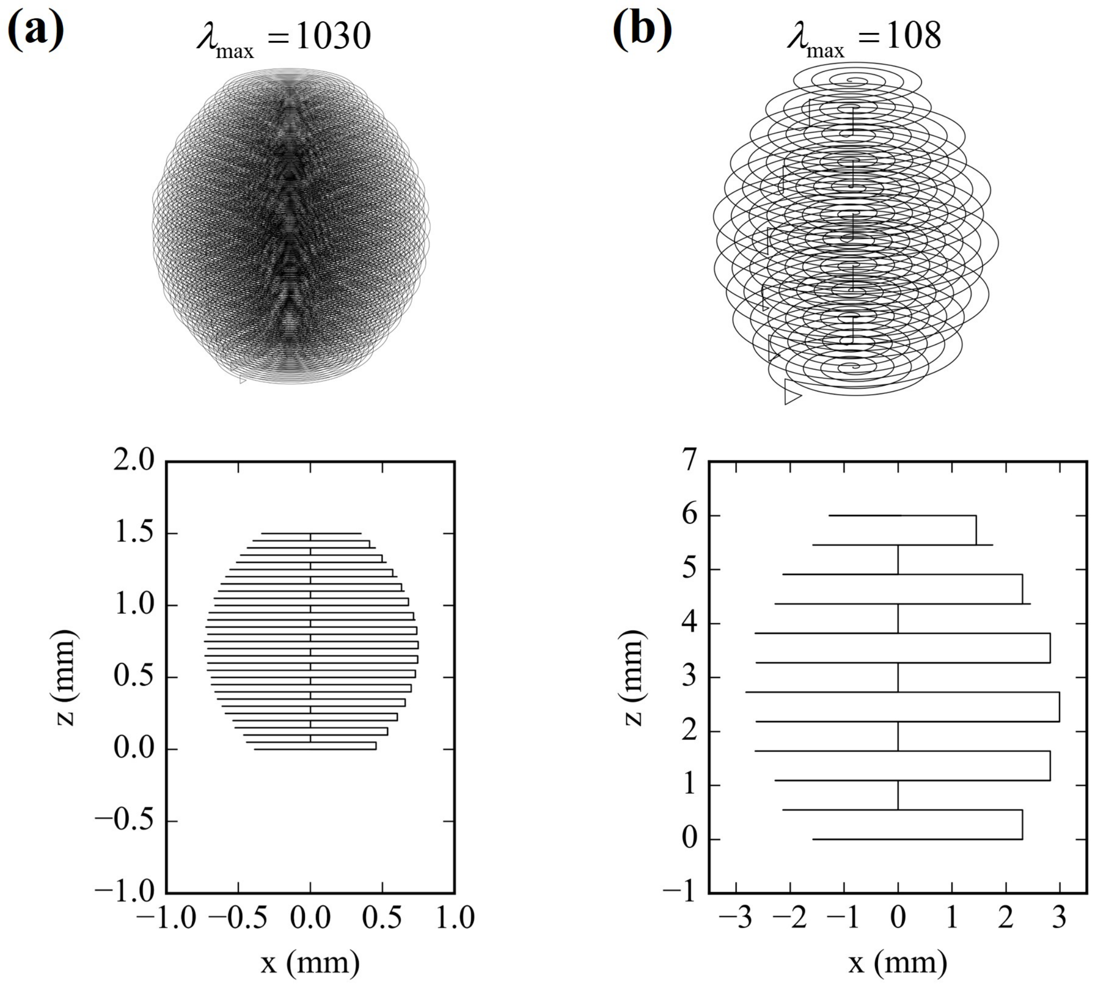

Figure S13: Spiral skein topology (a) $\lambda_{\max} = 1030$, (b) $\lambda_{\max} = 108$ generated using the design framework. Cross-sectional x–z projections show the layered spiral arrangement of fibers for two different skein dimensions. Each layer is formed by a spiral loop with alternating inward and outward winding, connected by vertical segments to create a continuous three-dimensional structure.

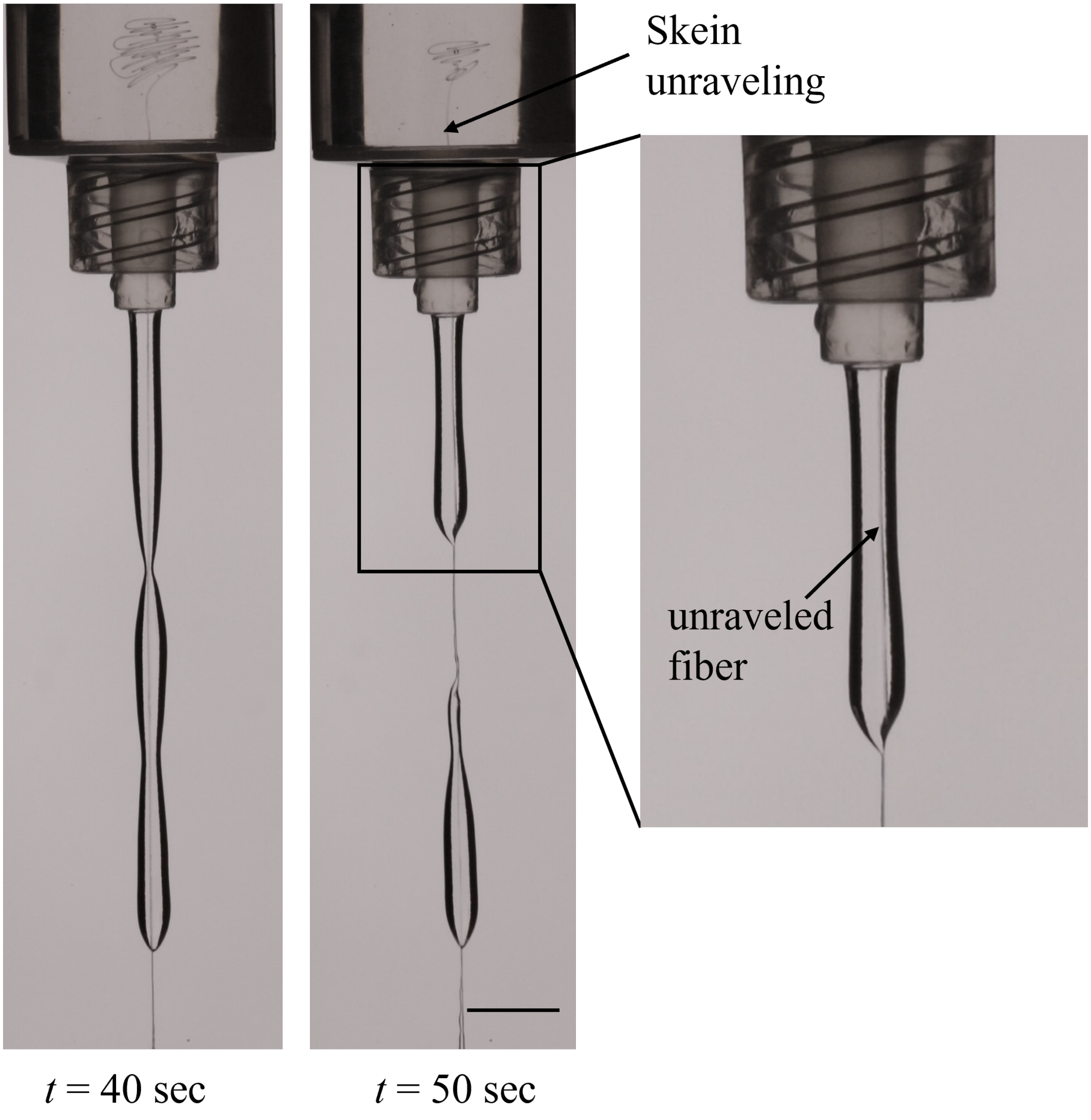

Figure S14: Time-lapse sequence showing the deployability of a synthetic skein under converging fluid flow in a yield stress fluid, $\sigma_y = 48$ Pa. The figure provides a close-up view at $t = 40$ s and $t = 50$ s, highlighting the unraveled fiber. Scale bar is 5 mm.

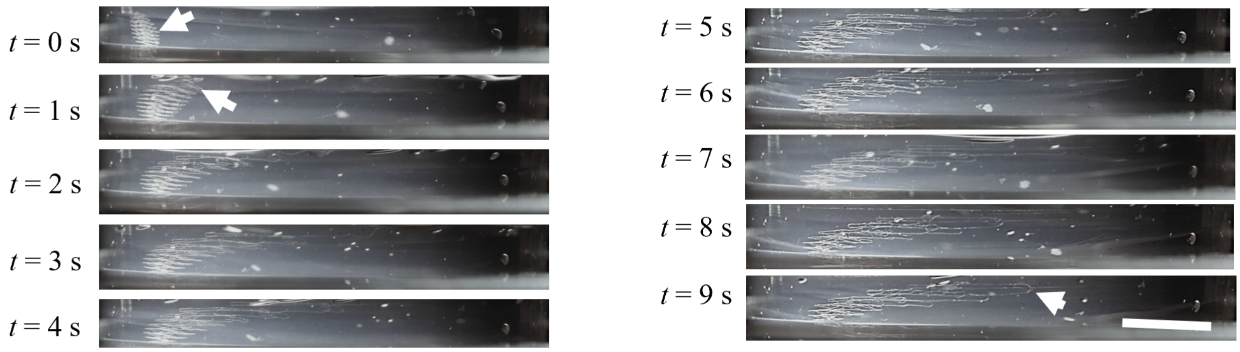

Figure S15: Side view of unraveling of a synthetic engineered skein with $\lambda = 131$, $D_o = 6$mm and $d_f \approx 200$ μm in simple shear flow with a rotational rheometer (DHR-3, TA Instruments) at $\dot{\gamma} = 1$ s^{-1}. The corresponding EP number is 50 with $\sigma_y = 48$ Pa, $\dot{\gamma}_{crit} = 9.3$, and $n = 0.4$. Scale bar is 1 cm.

Table S1: Comparison of hagfish skeins and engineered synthetic skeins with corresponding elastic modulus, fiber diameter, coil diameter, and hidden length ratio.

	E_f	d_f	D_o	λ
Hagfish skein	6 MPa	1–3 μm	100–150 μm	1000
Skein 1	5 MPa	30 μm	50 mm	123
Skein 2	23 MPa	3.5 μm	5 mm	131
Skein 3	23 MPa	3.5 μm	0.9 mm	131
Skein 4	23 MPa	3 μm	1.5 mm	1030

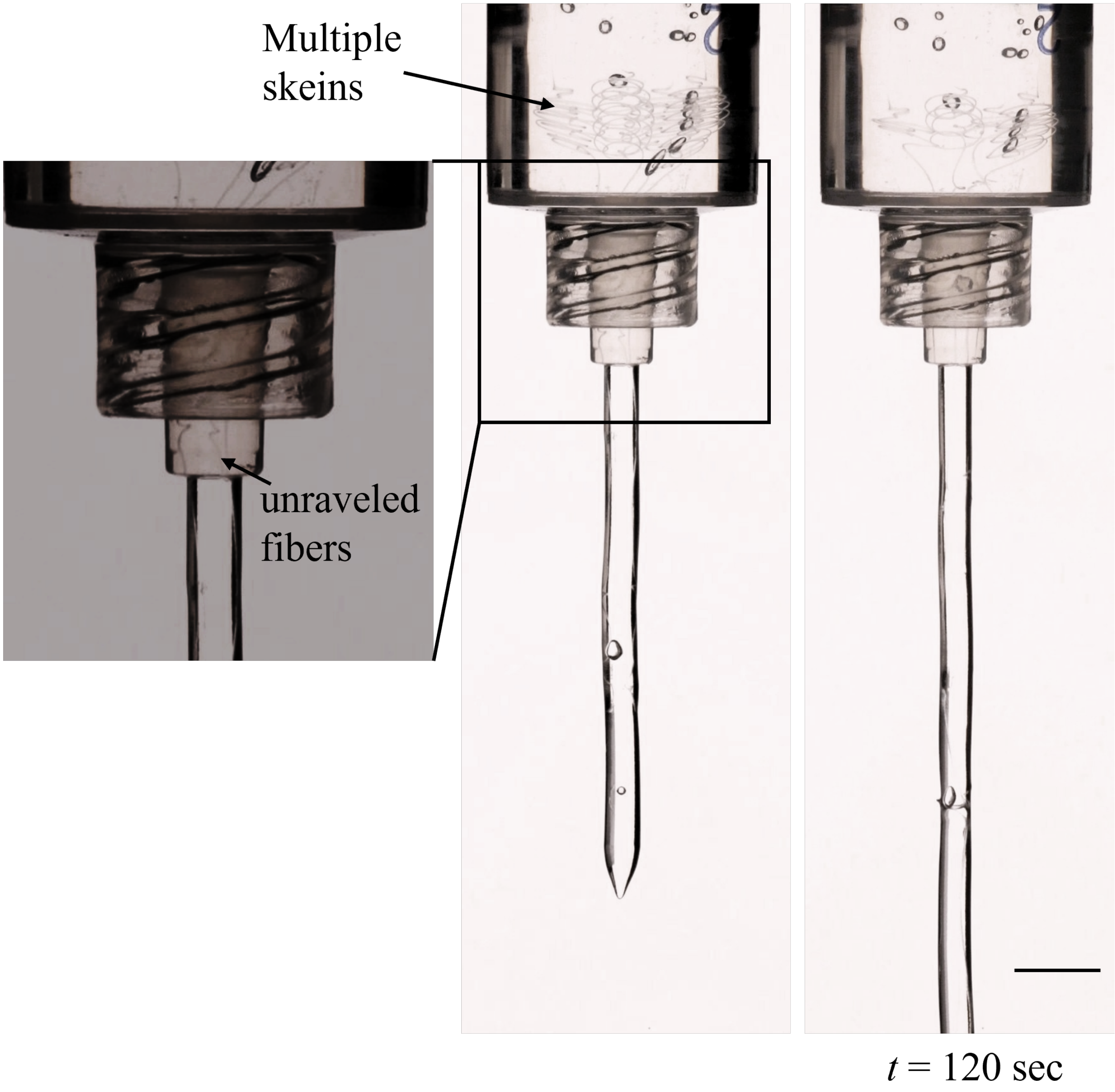

Figure S16: Gravity-driven extensional breakup suppressed by synthetic skeins. When multiple synthetic skeins (4) are embedded in the gel, their simultaneous unraveling resists extensional breakup, along with a close-up view highlighting the unraveled fibers. Scale bar is 5 mm.

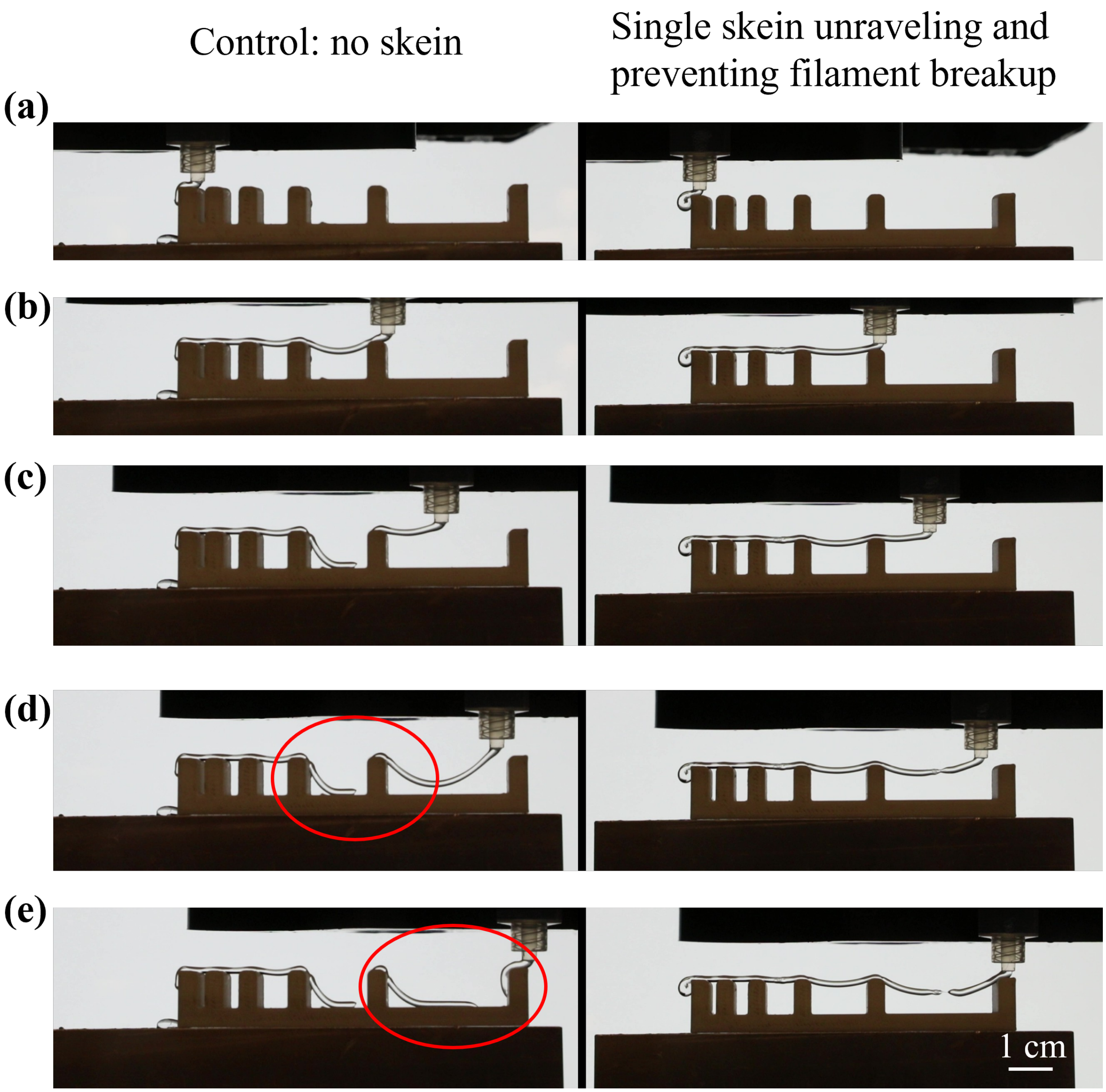

Figure S17: Demonstration of fibers unraveled during horizontal extrusion of a yield-stress fluid and the stabilizing effect of extended embedded fibers. (Left) Extrusion of yield stress fluid ($\sigma_y = 48$ Pa) in a gap spanning geometry, where the deposited filament quickly breaks when the applied stress exceeds the yield stress of the medium, preventing stable gap-spanning structures highlighted in the circled regions. (Right) Extrusion of yield stress fluid containing a synthetic skein with hidden length ratio $\lambda = 100$. As the skein unravels during extrusion, the unraveled fiber reinforces the printed structure and enables gap-spanning behavior.

References

[1] Wonsik Eom et al. “Fast 3D printing of fine, continuous, and soft fibers via embedded solvent exchange”. In: *Nature Communications* 16.1 (Jan. 2025), p. 842. ISSN: 2041-1723. DOI: 10.1038/s41467-025-55972-1. URL: https://doi.org/10.1038/s41467-025-55972-1.

[2] Randy H. Ewoldt and Chaimongkol Saengow. “Designing Complex Fluids”. In: *Annual Review of Fluid Mechanics* 54.Volume 54, 2022 (2022), pp. 413–441. ISSN: 1545-4479. DOI: 10.1146/annurev-fluid-031821-104935.

[3] Mohammad Tanver Hossain et al. “The critical plastocapillary number for a Newtonian liquid filament embedded into a viscoplastic fluid”. In: *Journal of Non-Newtonian Fluid Mechanics* (2025), p. 105440. ISSN: 0377-0257. DOI: 10.1016/j.jnnfm.2025.105440. URL: https://www.sciencedirect.com/science/article/pii/S037702572500059X.
